# Ultrasound-assisted Strecker synthesis of novel 2-(hetero)aryl-2-(arylamino)acetonitrile derivatives

**DOI:** 10.3762/bjoc.16.242

**Published:** 2020-11-30

**Authors:** Emese Gal, Luiza Gaina, Hermina Petkes, Alexandra Pop, Castelia Cristea, Gabriel Barta, Dan Cristian Vodnar, Luminiţa Silaghi-Dumitrescu

**Affiliations:** 1 Faculty of Chemistry and Chemical Engineering, Research Center on Fundamental and Applied Heterochemistry, Babes-Bolyai University, 11 Arany Janos street, RO-400028, Cluj-Napoca, Romania; 2 Department of Chemistry, Babes-Bolyai University, 11 Arany Janos street, RO-400028, Cluj-Napoca, Romania; 3 Department of Food Science and Technology, University of Agricultural Sciences and Veterinary Medicine 3-5 Mănăștur Street, RO-400372 Cluj-Napoca, Romania

**Keywords:** Ames test, α-aminoacetonitriles, ferrocene, phenothiazine, SEM, single crystal XRD, sonochemistry

## Abstract

This work describes an efficient, simple, and ecofriendly sonochemical procedure for the preparation of new α-(arylamino)acetonitrile derivatives C-substituted with phenothiazine or ferrocene units. The synthetic protocol is based on the Strecker reaction of a (hetero)aryl aldimine substrate with trimethylsilyl cyanide (TMSCN) in poly(ethylene glycol) (PEG) solution. The advantages of the sonochemical versus the conventional α-(arylamino)acetonitrile synthesis are the significantly shorter reaction time (30 min instead of 72 hours), the higher purity and the easier separation of the product that precipitated from the reaction mixture in crystalline form as depicted by scanning electron microscopy (SEM) analysis. The single crystal X-ray diffraction analysis disclosed the arrangement of the α-(arylamino)acetonitrile molecules in the aggregated crystalline state as a racemic mixture. The mutagenic/antimutagenic potential for three representative derivatives containing phenothiazinyl, ferrocenyl, and phenyl units, respectively, was evaluated by the Ames Salmonella/microsome test using *S. typhimurium* TA98 and TA100 strains with and without metabolic activation. The preliminary screening results pointed out that the C-(hetero)aryl-α-(arylamino)acetonitrile derivatives can be considered genotoxically safe and possibly antimutagenic.

## Introduction

Sonochemistry can be considered as a major contributor to green chemistry mainly due to the ability of minimizing the energy consumption required by chemical transformations and allowing the development of environmentally friendly chemical procedures which may be eventually scaled up for industrial applications [1]. For the synthesis of organic heterocyclic compounds, the use of ultrasound irradiation became a powerful tool by proving to be superior in terms of reaction rates, yields, and the purity of the products as compared to traditional convective heating methods [2]. Sonochemical syntheses can be successfully performed in homogeneous media using “green” solvents, for example, low vapor pressure solvents such as ionic liquids [3], low volatile solvents such as glycerol, ethylene glycol and its oligomers, or nontoxic water solvent, as well as in heterogeneous media under solvent-free conditions [4]. Other advantages induced by sonication are related to the possibility of controlling the crystal structure properties of the final product in nanomaterials syntheses [5]. For this reason, theoretical scientific research is currently directed towards the understanding of the physical phenomena involved in sonocrystallization mechanisms [6]. A major benefit of sonocrystallization appeared to be the induction of nucleation and therefore, crystallization improvements were operated for several organic compounds of low to medium molecular weight with the possibility of scaling up of this technology for industrial use [7]. Low ultrasonic frequencies between 20 and 100 kHz were reported in the literature as optimal to enhance the nucleation and fragmentation rates, but the exact optimal frequency is probably reactor and system specific [8].

The exciting properties of the heterocyclic phenothiazine core displaying tunable chemical, redox, optical, and biological properties upon a careful selection of the substitution pattern [9], continually encouraged the scientific community to search for new powerful representatives and to elaborate advantageous synthetic protocols. Under chemical, biochemical, or electrochemical conditions the ferrocene unit displays a remarkably reversible one-electron oxidation behavior offering interesting fields of applications for its derivatives as redox mediators in sensor applications [10]. Medicinal applications of ferrocene derivatives grafted on different pharmacophoric units greatly benefit from the lipophilic character of the metallocene [11]. However, only a limited number of reports described the application of ultrasonic irradiation for the synthesis of phenothiazine derivatives, and included the N-alkylation of 10*H-*phenothiazine [12], condensation of phenothiazine carbaldehyde with hydrazino-benzoxazole [13] or different other acetohydrazines [14], complexation of phenothiazinyl-chalcone using diiron nonacarbonyl [15], and regioselective oxidation [16].

α-Aminonitriles are versatile synthetic intermediates that are readily obtainable by a Strecker reaction involving the addition of a cyanide nucleophile to an imine C–N bond. Among the various cyanide transferring agents, which were largely documented in the search for advantageous synthetic procedures of these synthetic intermediates [17], trimethylsilyl cyanide (TMSCN) gave the possibility of accomplishing Strecker reactions using a wide variety of aldimine and ketoimine substrates under mild conditions [18]. Early studies reported a positive effect of sonication on the classical aminocyanation procedures. The reaction rate of the classical Strecker reaction using cyanide salts, amines and aromatic carbonyl derivatives [19] or piperidone [20] appeared improved under ultrasound-assisted conditions, which also enhanced the yields of the final α-aminonitrile derivatives. The Strecker reaction of cyclopropanone acetal substrates with sodium cyanide and several amines was also facilitated by sonication conditions which afforded cleaner N-alkylated α-(amino)cyanocyclopropane derivatives not contaminated by intermediates or ring-opening byproducts [21], whereas the asymmetric Strecker synthesis induced by chiral amines was successfully conducted by sonication in the presence of silica gel [22].

Pursuing our interest in developing environmentally friendly procedures for the synthesis of new phenothiazine and ferrocene derivatives [23–27] and perceiving the importance of α-aminoacetonitrile derivatives as pharmaceutical and agrochemical intermediates with a great number of α-aminonitrile derivatives which were proved to have remarkable biological properties exhibiting enzymatic activity as potent and selective protease inhibitors, fungicidal and herbicidal activity [17], we designed an efficient, simple, and ecofriendly synthetic procedure for the preparation of new synthetic compounds containing joint phenothiazine/ferrocene and α-amino-nitrile pharmacophoric units. In this work we report the experimental procedure for the ultrasound-assisted addition of the TMSCN nucleophile to heterocyclic aldimines. The substrates tested were mostly represented by a series of phenothiazinyl aldimines, but the scope of the new synthetic procedure was broadened by the preparation of new α-(arylamino)acetonitrile derivatives containing ferrocenyl and phenyl units. In order to evaluate the carcinogenic potential hazard of the newly prepared α-(arylamino)acetonitrile derivatives, the mutagenic potential of C-substituted derivatives containing phenothiazine, ferrocene or benzene units was screened by the *Salmonella* mutagenicity assay (Ames test) [28] using *S. typhimurium* TA98 and TA100 strains.

## Results and Discussions

### Ultrasound-assisted synthesis

**Scheme 1:**
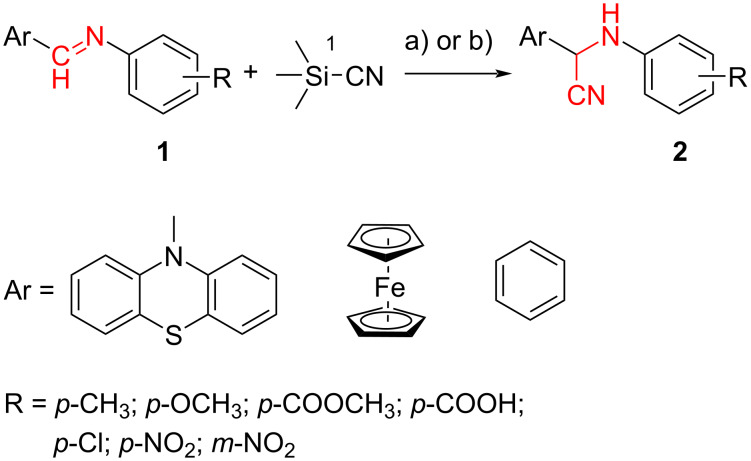
Synthesis of α-amino-acetonitrile derivatives. Reaction conditions: Aldimine (1 equiv), TMSCN (1 equiv), reaction medium PEG–H_2_O a) ultrasound-assisted reaction conditions: ultrasound frequency 37 kHz, power 95 W, temperature 25 °C, sonication time 30 min, b) classical reaction conditions: rt, 72 hours.

The Strecker reaction between (hetero)aromatic aldimines and TMSCN in poly(ethylene glycol) (PEG)–water medium [18] was customized for phenothiazinyl aldimine substrates (Scheme 1), but an extremely long reaction time was required (72 hours) for the reaction to complete. In order to enhance the reaction rate, alternative energy sources were taken into consideration and ultrasonic irradiation was selected as a green chemistry protocol capable of inducing a more efficient energy input. Indeed, the reaction rate was significantly increased by applying the new protocol modified by means of an indirect ultrasound irradiation technique and high yields of the α-(arylamino)acetonitrile products were obtained after 30 minutes of sonication in a commercially available ultrasonic bath. The ferrocene and phenothiazine derivatives behaved in a similar manner and excellent yields of the corresponding α-(arylamino)acetonitrile derivatives were obtained in each case (Table 1), indicative of a good tolerance for the presence of (hetero)aromatic units and either electron-donating (methyl, methoxy) or electron-withdrawing groups (carboxy, nitro, etc.) pending on the imine substrate.

**Table 1:** Synthesis and characterization of 2-arylamino-2-(hetero)arylacetonitrile derivatives.

entry	α-(arylamino)acetonitrile	yield (%)		mp (°C)	FTIR ν_C≡N_ (cm^−1^)

		classical	ultrasound		

	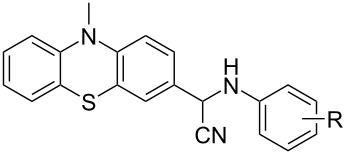				

**2a**	R = *p*-CH_3_	95	98	90	2245
**2b**	R = *p-*O-CH_3_	95	97	145	2247
**2c**	R = *p*-COOCH_3_	91	95	85	2230
**2d**	R = *p*-COOH	97	97	206	2260
**2e**	R = *p-*Cl	93	93	86	2260
**2f**	R = *p-*NO_2_	96	96	101	2234
**2g**	R = *m-*NO_2_	98	98	128	2231

	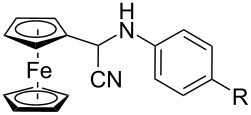				

**2h**	R = -O-CH_3_	90	92	85	2247
**2i**	R = -COOCH_3_	94	94	78	2250
**2j**	R = *p-*Cl	92	92	108	2239
**2k**	R = *p*-COOH	94	94	178	2239

	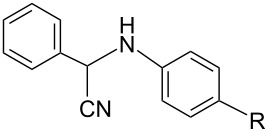				

**2l**	R = -COOCH_3_	94	95	129	2251

The molecular structures of all synthesized α-(arylamino)acetonitriles **2a–j** were fully characterized by spectroscopic methods. The MS spectra confirmed the molecular weight of the new compounds, while high-resolution ^1^H and ^13^C NMR spectroscopy afforded the complete characterization of the molecular skeleton. In the ^1^H NMR spectra of compounds **2c**, **2e–l** a vicinal coupling constant between the protons in the >CH–NH– unit (^3^*J* ≈ 7.5–8.7 Hz) was recorded suggesting the reduced mobility for the proton in the amino group. The presence of the cyano functional group was confirmed by the FTIR spectra displaying an absorption band typical for the stretching vibration of the C≡N bond situated in the 2230–2260 cm^−1^ region, its position appearing slightly influenced by the electronic effects induced by the substituents of the aniline unit (Table 1). Detailed information about characterization data for compounds **2a–l** is given in Supporting Information File 1.

### X-ray crystallographic data

**Figure 1:**
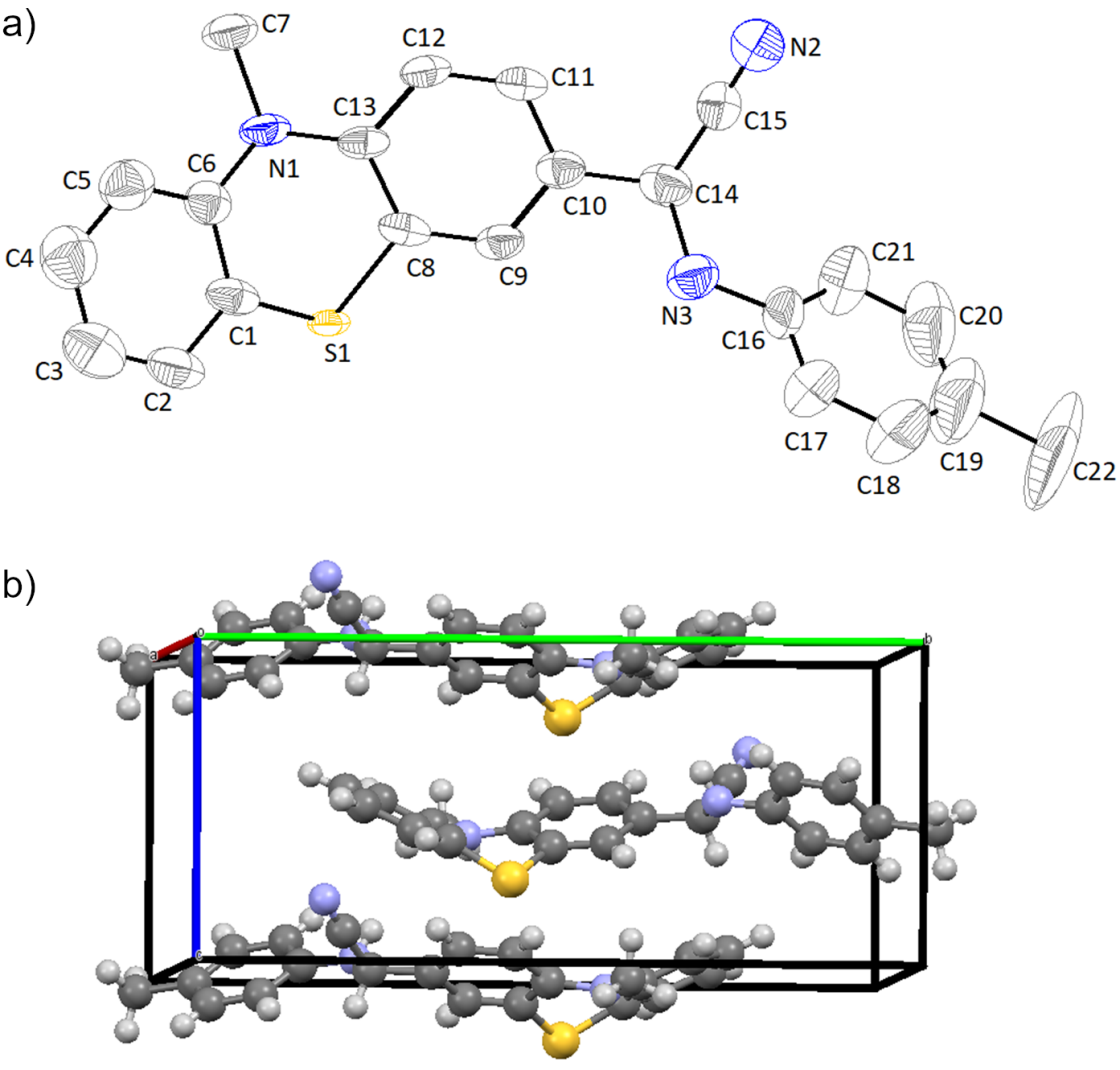
Crystal structure of 2-phenothiazinyl-2-(*p*-tolylamino)acetonitrile **2a**. a) ORTEP plot and b) crystallographic cell unit.

In order to bring evidence of the geometry and arrangement of the molecules of the synthesized α-(arylamino)acetonitrile derivatives in the aggregated crystalline state, single crystals suitable for X-ray analysis were obtained by crystallization from isopropanol. The crystallographic data for the structure **2a** reported in this paper have been deposited at the Cambridge Crystallographic Data Centre as supplementary publication (CCDC 2018198) (deposit@ccdc.cam.ac.uk). The molecular structure for the representative compound **2a** is depicted in Figure 1.

As revealed from the crystal packing structure shown in Figure 1, compound **2a** forms a single crystalline phase containing both enantiomers of the chiral molecular structure in an ordered 1:1 ratio in the elementary cell. The phenothiazine unit shows a quasi-equatorial orientation of the methyl group attached to the heterocyclic nitrogen atom and a folding angle of 143.3°, a value close to the typical folding angle for unsubstituted 10-methylphenothiazine which was reported to be 143.7° [29], thus revealing a negligible electron-withdrawing effect of the substituent. The aromatic plane situated on the edge of the phenothiazine and the plane of the aromatic unit attached to the amino substituent appear twisted with a dihedral angle of 64.5°. The intermolecular distances are situated in the range 2.3–2.6 Å disclosing the lack of intermolecular interactions in the crystal structure.

### Scanning electron microscopy (SEM) analysis

**Figure 2:**
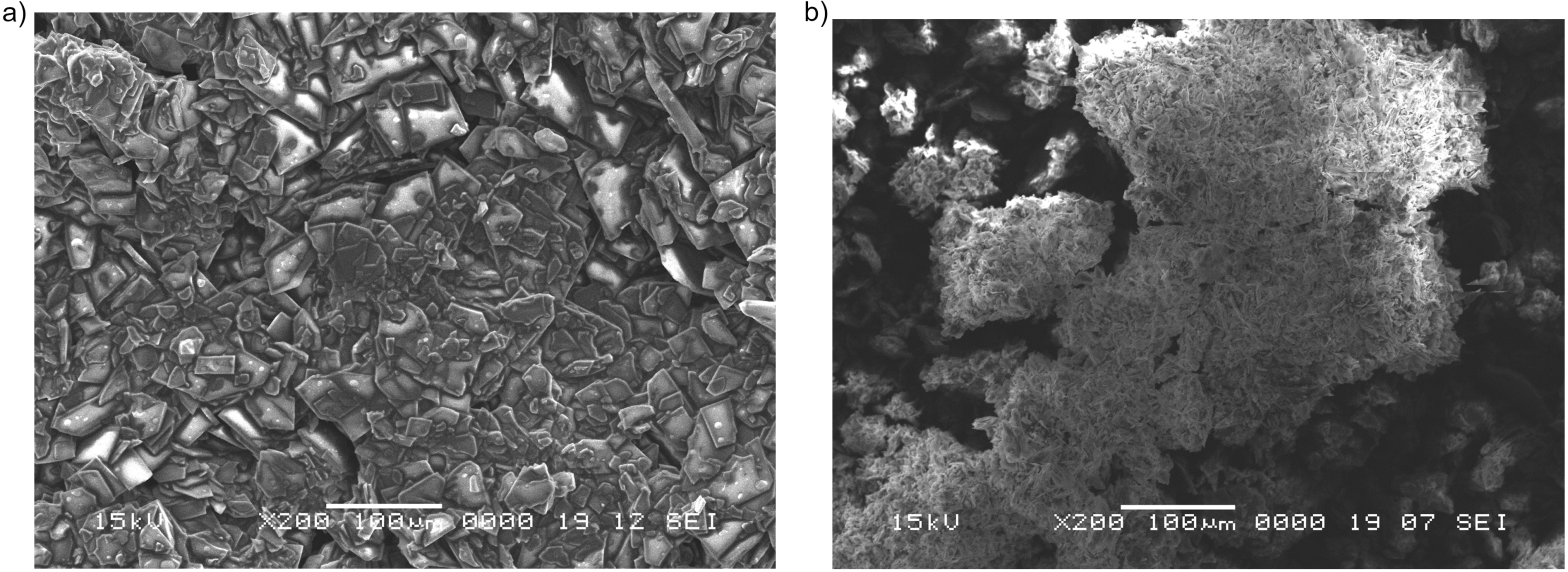
SEM images recorded at 200× for the raw reaction product **2b** obtained through a) ultrasound-assisted reaction conditions and b) classical reaction conditions.

**Figure 3:**
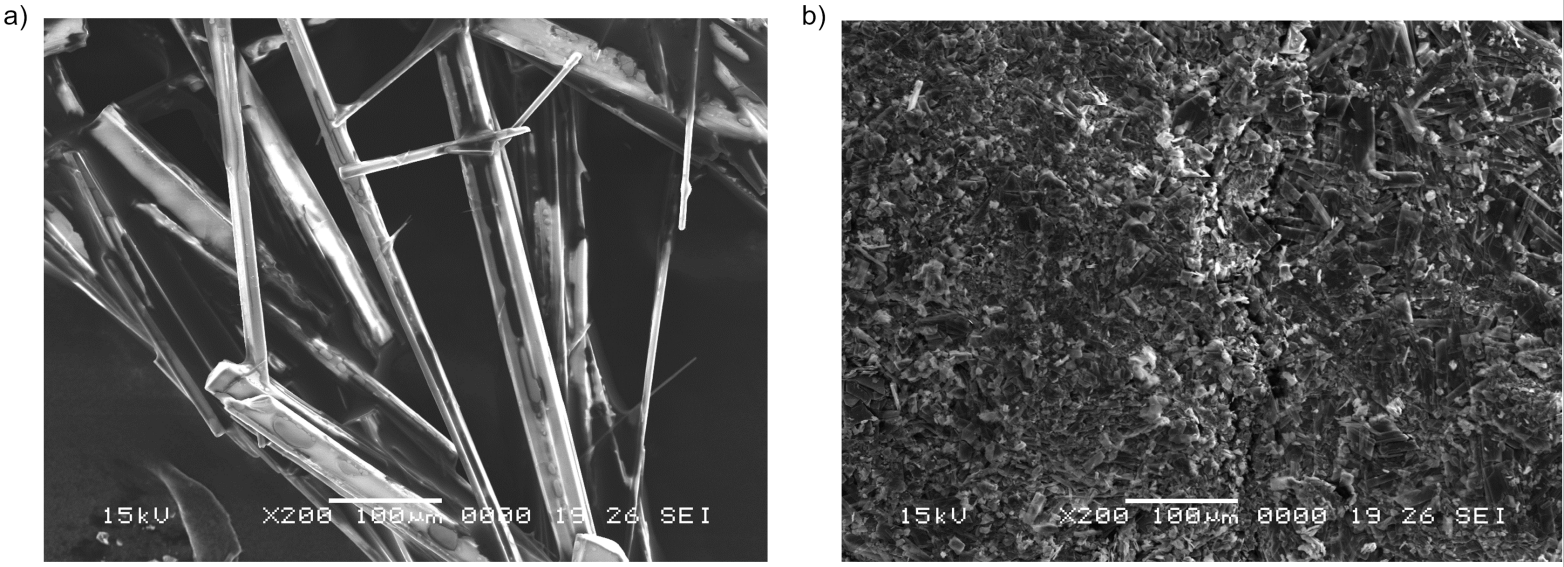
SEM image recorded at 200× for the raw reaction product **2c** obtained through a) ultrasound-assisted reaction conditions and b) classical reaction conditions.

Besides process intensification leading to a shorter reaction time, another advantage of the ultrasound-assisted reaction conditions was the formation of the product in crystalline form which was further investigated by SEM analysis. For comparison, the SEM micrographs recorded for the α-(arylamino)acetonitriles **2b** (Figure 2) and **2c** (Figure 3) obtained by ultrasound-assisted versus classical conditions are presented. In Figure 2a the magnification at 200× clearly shows higher crystallite sizes of the reaction product **2b** isolated by direct filtration of the reaction mixture obtained after the ultrasound-assisted protocol as compared to the same product obtained under classical conditions (Figure 2b).

In Figure 3a, the magnification at 200× indicates sharp rod-like natured crystals of the amino-acetonitrile **2c** obtained by the ultrasound-assisted reaction conditions in comparison with the crystallite aggregates obtained by the classical procedure (Figure 3b).

### Biological assay

We chose the well-recognized Ames test to screen the mutagenic potential for three selected C-substituted α-aminoacetonitriles comprising different hetero(aromatic) units: phenothiazinyl (**2c)**, ferrocenyl (**2i**), and phenyl (**2l**), respectively. The similarity of the tested aminoacetonitrile series was set aside by the N-functionalization of the methyl 4-((cyanomethyl)amino)benzoate skeleton, while dissimilarity was introduced by the C-functionalization with distinctive phenothiazinyl (**2c)**, ferrocenyl (**2i**), and phenyl (**2l**) units, respectively, each of the constituent structural units being susceptible of imparting biological activity. The viability of *Salmonella typhimurium* TA98 and TA 100, respectively, was assessed by exposing the histidine dependent bacteria to compound **2c**, **2i**, and **2l**, respectively, directly on minimal glucose agar plates in the presence or absence of the metabolic activation system S9. The number of histidine independent revertant colonies was scored on the test plates after 72 hours of incubation at 37 °C. The mutagenicity test results confirmed spontaneous colony numbers within the regular range (comparable to the number scored on the (negative) solvent control plates), thus indicating that no toxicity effects are predictable for the new compounds **2c**, **2i**, and **2l** at concentrations ranging at their solubility maxima.

The possible antimutagenic effect of the selected C-substituted α-aminoacetonitriles **2c**, **2i**, and **2l**, respectively, in the presence of 2-aminoantracene, daunomycin (mutagens for TA 98), or sodium azide (known mutagen for TA 100), was also examined using the plate incorporation procedure and the results are summarized in Table 2 and Table 3. This assay proved a strong antimutagenic activity of all tested α-(arylamino)acetonitrile derivatives, with a better inhibition exhibited in the case of the TA98 strain (44–95%), but nevertheless considerable for TA100 strain (46–79%).

**Table 2:** The antimutagenicity assay results of the newly synthesized C-substituted α-aminoacetonitriles for *S. typhimurium* TA98 and TA100 strain with the metabolic activation system S9.

test item	TA98		TA100	
	number of revertants	inhibition (%)	number of revertants	inhibition (%)

negative control	25		170	
**2c**	14	44	42	75.3
**2i**	10	60	35	79.4
**2l**	16	36	43	74.7
2-aminoanthracene^a^	28	–	114	–

^a^2-Aminoanthracene was used as the positive control for the *S. typhimurium* TA98 and TA100 strains.

**Table 3:** The antimutagenicity assay results of the newly synthesized C-substituted α-aminoacetonitriles for *S. typhimurium* TA98 and TA100 strain without the metabolic activation system S9.

test item	TA98		TA100	
	number of revertants	inhibition (%)	number of revertants	inhibition (%)

negative control	58		184	
**2c**	3	94.8	98	46.7
**2i**	14	75.8	38	79.3
**2l**	12	79.3	85	53.8
NaN_3_^a^	–	–	147	–
daunomycin^a^	95	–	–	–

^a^NaN_3_ and daunomycin were used as the positive controls for the *S. typhimurium* TA98 and TA100 strains.

The results of the antimutagenicity assay indicate a lower inhibition exhibited in the presence of liver homogenate (S9), pointing out that metabolizing enzymes could interfere with the activation of the synthetic compounds.

## Conclusion

An efficient reaction protocol for the ultrasound-assisted synthesis of new 2-(arylamino)-2-(hetero)arylacetonitrile derivatives containing phenothiazine units was developed. It was proved that the protocol can be more generally applied, affording excellent yields of the substituted α-(arylamino)acetonitrile derivatives with a high tolerance to the variation of (hetero)aromatic units and electron-donating/withdrawing substituents present in the structure of the aldimine substrate. The comparison between the classical and the ultrasound-assisted reaction protocols recommends the ultrasound irradiation technique as a greener chemical protocol affording significantly enhanced reaction rates and higher crystallite size of the products, thus increasing the energy efficiency of the Strecker synthesis.

An initial screening of the mutagenic potential of the newly synthetized α-amino-C-substituted-acetonitriles pointed out that the compounds **2c**, **2i**, and **2l** can be considered as genotoxically safe at concentrations in the range of their solubility limit in DMSO/water media. The tested compounds also exhibited antimutagenic activity by interfering with the effect of known mutagenic compounds such as 2-aminoantracene and sodium azide.

## Experimental

### General procedures for the preparation of 2-(arylamino)-2-(hetero)arylacetonitrile derivatives

#### Ultrasound-assisted reaction conditions (a)

Ultrasound-assisted reactions were carried out by indirect sonication using a commercially available ultrasonic bath (Elmasonic S 15 (H), Germany) of rectangular geometry (tank internal dimensions 151 × 137 × 100 mm) with sandwich transducer systems, an ultrasonic frequency of 37 kHz, power 95 W, and temperature-controlled ultrasonic operation. For the reactions, a stopper-sealed pear-shaped glass flask (25 mL) containing the reaction mixture was placed in the central position of the ultrasonic bath tank filled with a 5% Na_2_CO_3_ aqueous solution and subjected to sonication by setting the bath operation period to 30 min and the temperature to 25 °C. The reaction mixture was prepared by adding TMSCN (1 equiv) to a solution containing the aldimine (1 equiv) dissolved in PEG (5 mL) and water (1 mL). After completion of the reaction, the product was collected by filtration directly from the reaction mixture, or after being poured into water. The crystalline product was dried and if necessary, further purified by recrystallization from a suitable solvent.

#### Classical conditions (b)

The reaction mixture, prepared as described to general procedure (a), was stirred at room temperature for 3 days. After completion of the reaction, the mixture was poured into water and the product was extracted in diethyl ether. After evaporation of the organic solvent the solid product was collected and purified by recrystallization from a suitable solvent.

## Supporting Information

File 1 Experimental procedures, characterization data, biological assay, and copies of the ^1^H and ^13^C NMR spectra.
