# Supplementary material for: Ultrasound-assisted Strecker synthesis of novel 2-(hetero)aryl-2-(arylamino)acetonitrile derivatives
Source: Beilstein J Org Chem. 2020 Nov 30;16:2929–36. doi: 10.3762/bjoc.16.242 (PMC7722623; doi:10.3762/bjoc.16.242)
Supplement: File 1 — Experimental procedures, characterization data, biological assay, and copies of the 1H and 13C NMR spectra. [file Beilstein_J_Org_Chem-16-2929-s001.pdf]

BEILSTEIN JOURNAL OF ORGANIC CHEMISTRY

Supporting Information

for

Ultrasound-assisted Strecker synthesis of novel 2-(hetero)aryl-2-(arylamino)acetonitrile derivatives

Emese Gal, Luiza Gaina, Hermina Petkes, Alexandra Pop, Castelia Cristea, Gabriel Barta, Dan Cristian Vodnar and Luminița Silaghi-Dumitrescu

Beilstein J. Org. Chem. **2020,** *16,* 2929–2936. doi:10.3762/bjoc.16.242

Experimental procedures, characterization data, biological assay, and copies of the ^{1}H and ^{13}C NMR spectra

License and Terms: This is a supporting information file under the terms of the Creative Commons Attribution License (https://creativecommons.org/licenses/by/4.0). Please note that the reuse, redistribution and reproduction in particular requires that the author(s) and source are credited and that individual graphics may be subject to special legal provisions.
The license is subject to the *Beilstein Journal of Organic Chemistry* terms and conditions: (https://www.beilstein-journals.org/bjoc/terms)

Table of contents

Experimental procedures and characterization data for compounds **2a**–**l**

XRD data for compound **2a**

Biological assay procedures

Copies of ^{1}H NMR and ^{13}C NMR spectra

Experimental

1. Materials and apparatus

The starting Schiff bases **1a–j** were prepared by the microwave-assisted condensation of the corresponding (hetero)aromatic aldehydes with aromatic primary amines according to our previously reported procedure [1]. Commercial reagent grade TMSC and the PEG solvent were purchased from Sigma Aldrich and employed without further purification. Thin layer chromatography was performed on Merck DC Alufolien, silica gel 60 F_{254}, and components were visualized by UV VL-4LC. The melting points are uncorrected and were determined in capillaries with an Electrothermal 9100 instrument. All reactions were carried out in an ultrasonic bath Elmasonic S 15 (H), Elma Schmidbauer GmbH, Gottlieb-Daimler-Str. 17, D-78224 Singen, Germany.

2. Spectral measurements

HRMS spectra were recorded using a Thermo LTQ Orbitrap XL instrument.NMR spectra were recorded at room temperature on a 600 MHz Bruker Avance instrument. Chemical shifts are expressed in terms of δ (ppm), relative to the standard tetramethylsilane (TMS). FTIR spectra were recorded using an ATR Bruker Vector 22 instrument. A digital Polarimeter, Perkin Elmer 341 was used for the measurement of the optical rotation angle. The experiment was conducted at room temperature using the electromagnetic radiation with λ=589.3 nm (sodium D-line). The sample contained a solution (0.5 mg/mL) of α-(arylamino)acetonitrile **2a** in ethanol, placed in a 100 mm tube.The SEM measurements were performed using a JEOL JSM 5600 LV microscope, equipped with an EDX spectrometer, Oxford Instruments (INCA 200 software). The energy of the acceleration beam employed was 15 kV and the given results are 200× magnifications. The

crystallographic data were collected on a Bruker SMART APEX diffractometer by using graphite-monochromatic MoKα radiation (λ = 0.71073 Å) at room temperature (294 K). The structures were refined with anisotropic thermal parameters and the hydrogen atoms were refined with a riding model and a mutual isotropic thermal parameter. For structure solving and refinement the software package SHELX-97 was used [2]. The drawings were created with the Diamond program [3]. Crystallographic data (excluding structure factors) for the structure **2a** in this paper have been deposited with the Cambridge Crystallographic Data Centre as supplementary publication no. 2018198 CCDC. Copies of the data can be obtained, free of charge, on application to CCDC, 12 Union Road, Cambridge CB2 1EZ, UK, (fax: +44-(0)1223-336033 or by email: deposit@ccdc.cam.ac.uk).

3. General procedure for the preparation of α-arylamino-acetonitrile derivatives

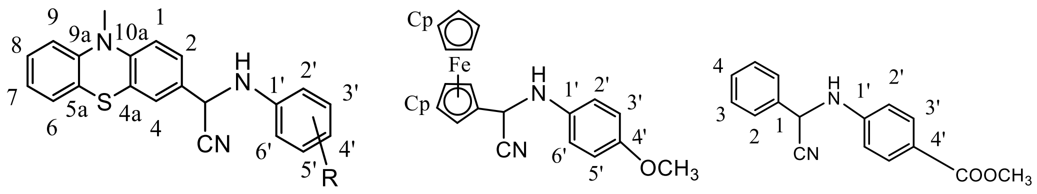

a) Ultrasound-assisted reaction conditions

The reaction mixture prepared by adding TMSCN (1 equiv) to a solution containing the aldimine (1 equiv) dissolved in PEG (5 mL) and water (1 mL), was placed in a 25 mL beaker and sonicated (37 kHz, 95 W) for 30 minutes at 25 °C. After completion of the reaction, the product was collected by filtration directly from the reaction mixture, or after being poured into water. The crystalline product collected by filtration was dried and, if required, further purification can be performed by recrystallization.

b) Classical conditions

The reaction mixture, prepared according to the procedure described above (a), was stirred at room temperature for 3 days. After completion of the reaction, the mixture was poured into water and the product was extracted in diethyl ether. After evaporation of the organic solvent, the solid product was purified by recrystallization.

2-(10-Methyl-10*H*-phenothiazin-3-yl)-2-(*p*-tolylamino)acetonitrile (**2a**)

Recrystallization from isopropanol gave a light yellow solid, yield: 95 % (0.30 g); m.p. 94-95 ^{0}C; FT-IR (KBr, $\tilde{\upsilon}_{max}$/cm^{-1}) 3357 (ν_{N-H}, m), 2991, 2959, 2835 (ν_{C-H}, m), 2231 ($\nu_{C≡N}$, w). HRMS (ESI): m/z calcd. for $C_{22}H_{19}N_3S$ [M$^+$], 357.1294; found 357.1295; ^{1}H NMR (600 MHz, $CDCl_3$) δ ppm: 2.30 (s, 3H,CH_3), 3.40 (s, 3H, N-CH_3), 3.89 (br, 1H, NH), 5.31 (s, 1H, C<u>H</u>CN), 6.98 (d,

2H, J =8.4Hz, $H_{Ph2'}$), 6.83-6.86 (m, 2H, H_1, H_9), 6.99 (td, 1H, J=7.5Hz, J=0.9Hz, H_7), 7.10 (d, 2H, J=8.4Hz, $H_{Ph3'}$), 7.16 (dd, 1H, J=7.6Hz, J=1.3Hz, H_6), 7.22 (td, 1H, J=7.8Hz, J=1.3Hz, H_8), 7.36 (d, 1H, J=1.9Hz, H_4), 7.39 (dd, 1H, J=8.4Hz, J=1.9, H_2); ^{13}C NMR (150 MHz, $CDCl_3$) δ ppm: 20.5 (CH_3), 35.4 (NCH_3), 49.9 (CH), 114.2 (C_1), 114.3 (C_9), 114,5 ($2C_{2'}$), 118.3 (q CN), 122.6 (q C_{4a}), 122.9 (C_7), 124.7 (q C_{5a}), 125.8 (C_6), 126.8 (C_8), 127.2 (C_4), 127.7 (C_2), 128.0 (q $C_{4'}$), 129.7 (q C_3), 130 ($2C_{3'}$), 142.3 (q $C_{1'}$), 145.1(q C_{10a}), 146.8(q C_{9a}).

2-(4-Methoxyphenylamino)-2-(10-methyl-10*H*-phenothiazin-3-yl)acetonitrile (2b)

Recrystallization from ethyl acetate gave a yellow solid, yield: 95 % (0.30g); m.p. 144-145 °C; FT-IR (KBr, $\tilde{\upsilon}_{max}$/cm^{-1}) 3350 (ν_{N-H}, m) 2960, 2832 (ν_{C-H}, m), 2233 (νC≡N, w), 1033 (ν_{C-O}, m). HRMS (APCI): m/z calcd. for $C_{21}H_{19}N_2SO$ $[M-CN]^+$, 347.1218; found $[M-CN]^+$ 347.1236. ^1H NMR (600 MHz, $CDCl_3$) δ ppm: 3.40 (s, 3H, N-CH_3), 3.76 (br, 1H, NH), 3.79 (s, 3H,OCH_3), 5.26 (s, 1H, CNCH), 6.76 (d, 2H, J =8.8Hz, $H_{Ph2'}$), 6.83-6.87 (m, 4H, $H_{Ph3'}$, H_1, H_9), 6.99 (td, 1H, J=7.5Hz, J=1.0Hz, H_7), 7.17 (dd, 1H, J=7.5Hz, J=1.2Hz, H_6), 7.23 (td, 1H, J=7.5Hz, J=1.3Hz, H_8), 7.36 (d, 1H, J=1.9Hz, H_4), 7.39 (dd, 1H, J=8.4Hz, J=1.9, H_2); ^{13}C NMR (150 MHz, $CDCl_3$) δ ppm: 35.4 (NCH_3), 50.7 (CH), 55.6 (OCH_3), 114.2 (C_1), 114.3 (C_9), 115.0($2C_{3'}$), 116.3($2C_{2'}$), 118.4 (q CN), 122.6 (q C_{4a}), 122.9 (C_7), 124.7 (q C_{5a}), 125.8 (C_6), 126.4 (C_8), 127.2 (C_4), 127.7 (C_2), 128.1(q C_3), 138.5 (q $C_{1'}$), 145.1(q C_{10a}), 146.8 (q C_{9a}), 154.1(q $C_{4'}$).

Methyl 4-((cyano(10-methyl-10*H*-phenothiazin-3-yl)methyl)amino)benzoate (2c)

Recrystallization from ethanol gave an orange solid, yield: 91 % (0.29g); m.p. 253 ^{0}C decomp.; FT-IR (KBr, $\tilde{\upsilon}_{max}$/cm^{-1}) 3340 (ν_{N-H}, w), 2975, 2870, 2135 ($\nu_{C\equiv N}$, w), 1680 ($\nu_{C=O}$, s). HRMS (APCI): m/z calcd. for $C_{22}H_{19}N_2SO_2$ $[M-CN]^+$, 375.1167; found $[M-CN]^+$ 375.1170. ^1H NMR (600 MHz, $CDCl_3$) δ ppm: 3.20 (s, 3H, N-CH_3), 3.81 (s, 3H, O-CH_3), 4.83 (br, 1H, NH), 5.38 (d, 1H, J=7.7Hz, CNCH), 6.68 (d, 2H, J=8.4Hz, $H_{Ph2'}$), 6.74 (d, 1H, J=8.2, H_1), 6.78 (d, 1H, J=8.1, H_9), 6.91 (t, 1H, J=7.5Hz, H_7), 7.07 (d, 1H, J=7.5Hz, H_6), 7.14(t, 1H, J=7.5Hz, H_8), 7.27-7.28 (m, 2H, H2, H4), 7.85(d, 2H, J=8.4Hz, $H_{Ph3'}$). ^{13}C NMR (150 MHz, $CDCl_3$) δ ppm: 35.3 (NCH_3), 48.3 (CH), 51.7 (OCH_3), 112.9 (2 $C_{2'}$), 114.3 (C_1), 114.4 (C_9), 117.7 (q CN), 120.8 (q$C_{4'}$), 122.3 (q C_{4a}), 122.9 (C_7), 124.6 (q C_{5a}), 125.6 (C_6), 126.4 (C_8), 127.1 (C_4), 128.8(C_2, q C_3), 131.8($2C_{3'}$), 145.0 (q C_{10a}), 146.7 (q C_{9a}), 148.7 (q $C_{1'}$), 167.0 (q C=O).

4-((Cyano(10-methyl-10*H*-phenothiazin-3-yl)methyl)amino)benzoic acid (2d)

Recrystallization from isopropanol gave a light brown solid, yield: 97 % (0.31g); m.p. 172-174 ^{0}C, FT-IR (KBr, $\tilde{\upsilon}_{max}$/cm^{-1}) 3440 (ν_{N-H}, w), 3081 (ν_{O-H}, broad), 2962, 2867, 2874, 2242 ($\nu_{C\equiv N}$, w), 1696 ($\nu_{C=O}$, s); HRMS (ESI): m/z calcd. for $C_{22}H_{18}N_3SO_2$ [M+1] $^{+}$ 388.1120; found 388.2554; ^{1}H NMR (600 MHz, CD_3COCD_3) δ ppm: 3.40 (s, 3H, CH_3), 5.91(s, 1H, CNCH), 6.6 (br, 1H, NH), 6.93-7.03 (m, 5H, H_7, $H_{Ph2'}$), 7.16(d, 1H, J=7.2Hz, H_6), 7.23(t, 1H, J=7.4Hz, H_8), 7.39 (s, 1H, H_4), 7.48(d, 1H J=7.8Hz, H_2) 7.91(d, 2H, J=8.2Hz, $H_{Ph3'}$). ^{13}C NMR (150 MHz, $CDCl_3$) δ ppm: 34.9 (NCH_3), 47.7(CH) 112.8(2$C_{2'}$), 114.6(C_1, C_9), 118.3 (q CN), 120.5 (q $C_{4'}$), 122.2 (q C_{4a}), 122.8 (C_7), 123.9 (q C_{5a}), 125.6 (C_6), 126.8 (C_8), 126.9(C_4), 127.9 (C_2), 128.5 (q C_3), 131.4(2$C_{3'}$), 145.3 (q C_{10a}), 146.6 (q C_{9a}), 148.7 (q $C_{1'}$), 167.0 (q C=O).

2-(4-Chlorophenylamino)-2-(10-methyl-10*H*-phenothiazin-3-yl)acetonitrile (2e)

Recrystallization from ethyl acetate gave a light yellow solid, yield: 93 % (0.29g); m.p. 206-207 ^{0}C; FT-IR (KBr, $\tilde{\upsilon}_{max}$/cm^{-1}) 3335 (ν_{N-H}, w), 2969, 2873, 2228 ($\nu_{C\equiv N}$, w). HRMS (ESI): m/z calcd. for $C_{20}H_{16}ClN_2S$ [M-CN]$^{+}$ 351.0717; found 351.0741; ^{1}H NMR (600 MHz, $CDCl_3$) δ ppm: 3.41 (s, 3H, CH_3), 4.03(d, 1H, J=8.0Hz, NH), 5.30 (d, 1H, CNCH, =8.0Hz), 6.70 (d, 2H, J =8.8Hz, $H_{Ph2'}$), 6.84-6.87 (m, 2H, H_1, H_9), 6.99 (td, 1H, J=7.6Hz, J=0.8Hz, H_7), 7.17 (dd, H, J=7.6Hz, H_6), 7.21-7.24(m, 2H, H_8, $H_{Ph3'}$), 7.34 (d, 1H, J=2.1Hz, H_4), 7.38 (dd, 1H, J=8.3Hz, J=2.1, H_2); ^{13}C NMR (150 MHz, $CDCl_3$) δ ppm: 35.4 (NCH_3), 49.5 (CH), 114.33 (C_1), 114.39 (C_9), 115.3(2$C_{2'}$), 117.8 (q CN), 122.5 q C_{4a}, 123.0 (C_7), 125.0 (qC_{5a}), 125.1 (q $C_{4'}$), 125.7 (C_6), 126.3 (C_8), 127.2 (C_4), 127.3 (q C_3), 127.8 (C_2), 129.4(2$C_{3'}$), 143.1 (q C_{10a}), 145.0 (q C_{10a}), 147.0 (q $C_{1'}$).

2-(10-Methyl-10*H*-phenothiazin-3-yl)-2-(4-nitrophenylamino)acetonitrile (2f)

Purified by recrystallization from isopropanol, then from ethyl acetate gave a yellowish-brown solid, yield: 96% (0.30g); m.p. 151-152 ^{0}C ; FT-IR (KBr, $\tilde{\upsilon}_{max}$/cm^{-1}) 3378 (ν_{N-H}, w), 2956, 2819, 2238 ($\nu_{C\equiv N}$, w), 1531, 1352 ($\nu_{as,sim\ NO}$, s). HRMS (APCI): m/z calcd. for $C_{20}H_{16}N_4SO_2$ [M-CN]$^{+}$, 362.0963; found [M-CN]$^{+}$ 362.0964. ^{1}H NMR (600 MHz, $CDCl_3$) δ ppm: 3.31(s, 3H, NCH_3), 5.07 (br, 1H, NH), 5.45(1H, d, J=7.2Hz, CH), 6.69 (d, 2H, J =9.1Hz, $H_{Ph2'}$), 6.77 (d, 1, J=8.1Hz, H_1), 6.78 (d, 1, J=8.4Hz, H_9), 6.91 (t, 1H, J=7.5Hz, H_7), 7.07(dd, 1H, J=7.5Hz, J=1.3Hz, H_6), 7.14 (td, 1H, J=8.4 Hz, J=1.3HZ, H_8), 7.25 (d, 1H, J=2.0Hz, H_4), 7.30 (dd, 1H, J=8.1Hz, J=2.0, H_2), 8.04(d, 2H, J =9.1Hz, $H_{Ph3'}$). ^{13}C NMR (150 MHz, $CDCl_3$) δ ppm: 35.4 (NCH_3), 48.1 (CH), 112.7 (2$C_{2'}$), 114.2 (C_1), 114.4 (C_9), 117.2 (qCN), 122.3 (qC_{4a}), 123.0 (C_7), 124.8 (qC_{5a}), 125.6

6 (C_6), 126.0(2$C_{3'}$), 126.4 (C_8), 126.5 (q C_3), 127.1 (C_2), 127.8 (C_4), 139.7 (q $C_{4'}$), 144.9 (q C_{9a}), 149.9 (q C_{10a}), 150.3 (q $C_{1'}$).

2-(10-Methyl-10*H*-phenothiazin-3-yl)-2-(3-nitrophenylamino)acetonitrile (2g)

Recrystallization from isopropanol, then from ethyl acetate gave a brown solid, yield: 98 % (0.31g); m.p. 138-139 ^{0}C; FT-IR (KBr, $\tilde{\upsilon}_{max}$/cm^{-1}) 3378 (ν_{N-H}, m), 2956, 2819, 2238 ($\nu_{C\equiv N}$, w), 1531, 1352 ($\nu_{as,sim\ NO}$, s); HRMS (APCI): m/z calcd. for $C_{20}H_{16}N_4SO_2$ [M-CN]$^+$, 362.0963; found [M-CN]$^+$ 362.096; ^{1}H NMR (400 MHz, $CDCl_3$), δ ppm: 3.40 (s, 3H, CH_3), 4.47 (d, 1H, J=7.8Hz, NH), 5.40 (d, 1H, J=7.8Hz, CH), 6.85 (d, 2H, J=8.4Hz, H_1, H_9), 6.97-7.04 (m, 2H, $H_{Ph6'}$, H_7), 7.15 (dd, 1H, J=7.5Hz, J=1.1, H_6), 7.22 (t, 1H, , J=7.5Hz, J=1.1Hz, H_8), 7.59 (d, 1H, J=1.8Hz, H_4), 7.37-7.43 (m, 2H, H_2, $H_{Ph5'}$), 7.59 (s, 1H, $H_{Ph2'}$), 7.72 (d. 1H, J=8.0Hz, J-1.1Hz, $H_{Ph4'}$,); ^{13}C NMR (100 MHz, $CDCl_3$), δppm: 35.4 (NCH_3)**,** 49.0 (CH), 108.3 ($C_{2'}$), 114.42 (C_1), 114.45 (C_9), 114.8 ($_{4'}$), 117.3(CN), 119.6 ($C_{6'}$), 122.4 (qC_{4a}), 123.1(C_7), 125.1 (qC_{5a}), 125.7 (C_6), 126.4 (C_8), 126.6 (q C_3), 127.2 (C_2), 127.8 (C_4), 130.0 ($C_{5'}$), 144.9 (q C_{9a}), 145.3 (q C_{10a}), 147.2 (q$C_{1'}$), 149.2 (q $C_{3'}$).

2-(4-Methoxyphenylamino)-2-(ferrocenyl)acetonitrile (2h)

Recrystallization from ethanol gave a brown solid, yield: 90 % (0.29g); m.p. 107-108 ^{0}C; FT-IR (KBr, $\tilde{\upsilon}_{max}$/cm^{-1}) 3328 (ν_{N-H}, w) 3103 (ν_{O-H}, m), 2955, 2829, 2225 ($\nu_{C\equiv N}$, w), 1510, 1106 ($\nu_{C=C}$, s), 1463 (ν_{C-O}, m); HRMS (APCI): m/z calcd. for $C_{18}H_{18}$ Fe NO [M-CN]$^+$, 320.0738; found [M-CN]$^+$ 320.1636; ^{1}H NMR (600 MHz, $CDCl_3$) δ ppm 3.80 (s, 3H, OCH_3), 3.85(br, 1H, NH), 4.31-4.33 (m, 7H, H-Cp), 4.43 (s , 1H, H-Cp), 4.49 (s , 1H, H-Cp), 5.06 (s, 1H, CNCH), 6.78-88 (m, 4H, H_{Ph}); ^{13}C NMR (150 MHz, $CDCl_3$) δ ppm: 47.8 (CH), 55.7 (OCH_3), 66.8 (C_{Cp}), 68.2(C_{Cp}), 68.9(C_{Cp}), 69.1(C_{Cp}), 69.3(5C overlap), 82.6 (q C_{Cp}) , 115.0(2$C_{Ph3'}$), 116.2(2$C_{Ph2'}$), 118.6 (CN), 138.7 (q $C_{1'}$), 151.0 (q $C_{4'}$).

Methyl 4-(cyano(ferrocenyl)methylamino)benzoate (2i)

Recrystallization from ethanol gave a light brown solid, m.p. 140-141 ^{0}C, yield: 94 % (0.30g); FT-IR (KBr, $\tilde{\upsilon}_{max}$/cm^{-1}) 3329 (ν_{N-H}, s), 2946, 2905, 2869, 2246 ($\nu_{C\equiv N}$, w), 1669 ($\nu_{C=O}$, s), 1523, 1087 ($\nu_{C=C}$, s), 1352 (ν_{C-O}, m); HRMS (APCI): m/z calcd. for $C_{19}H_{18}FeNO_2$ [M-CN]$^+$, 348.0687; found [M-CN]$^+$ 348.0697; ^{1}H NMR (400 MHz, DMSO-d_6), δ (ppm): 3.88(s, 3H, $COOCH_3$), 4.31-4.33(m, 7H, H-Cp), 4.42(s, 6H, H-Cp) 4.47 (s, 1H, H-Cp), 4.69 (d, 1H, J=7.9Hz, NH), 5.19 (d, 1H, J=7.9Hz, CNCH), 6.74 (d, 2H, J=8.6Hz, $H_{Ph2'}$), 7.97 (d, 2H, J=8.6Hz, $H_{Ph3'}$); ^{13}C NMR (100 MHz, DMSO-d_6), δ ppm: 45.6 (CH), 51.8(OCH_3), 66.8 (C_{Cp}), 68.3 (C_{Cp}), 69.2 (C_{Cp}), 69.4,

69.5 (5C_{Cp}, overlap), 81.5 (q C_{Cp}), 112.6 (2$C_{2'}$), 117.6 (q CN), 121.1 (q $C_{4'}$), 131.7(2$C_{3'}$), 148.4 (q $C_{1'}$), 166.9 (q CO).

2-(4-Chlorophenylamino)-2-(ferrocenyl)acetonitrile (2j)

Recrystallization from ethanol gave a brown solid, m.p. 107-108°C, yield 92% (0.3g); FT-IR (KBr, $\tilde{\upsilon}_{max}$/cm^{-1}) 3313 (ν_{N-H}, s), 2915, 2869 (ν_{C-H}, m), 2239 ($\nu_{C\equiv N}$, w), 1509, 1077 ($\nu_{C=C}$ s);HRMS (ESI): m/z calcd. for $C_{17}H_{15}$ ClFe N $[M-CN]^+$, 324.0242; found $[M-CN]^+$ 324.0363; ^{1}H NMR (600 MHz, $CDCl_3$): δ (ppm) 4.18 (br, 1H, NH), 4.46(s, 1H, H-Cp), 4.41(s, 1H, H-Cp) 4.32 (s, 7H, H-Cp), 5.09 (d, 1H, J=6.4Hz, CNCH), 6.70-6.71 (m, 2H, $H_{Ph2'}$), 7.23-7.24 (m, 2H, $H_{Ph3'}$); ^{13}C NMR (150 MHz, $CDCl_3$), δ ppm: 46.5 (CH), 66.8(C_{Cp}), 68.3(C_{Cp}), 69.1(C_{Cp}), 69.3(C_{Cp}), 69.4(5C_{Cp}, overlap), 81.9 (qC_{Cp}), 115.1(2$C_{2'}$), 118.1 (q CN), 124.7 (q $C_{4'}$), 129.4 (2$C_{3'}$), 142.3 (q $C_{1'}$).

4-((Cyano(ferrocenyl)methylamino)benzoic acid (2k)

Recrystallization from ethanol gave a dark brown solid, m.p. 177-178°C, yield 94%, (0.3g); FT-IR (KBr, $\tilde{\upsilon}_{max}$/cm^{-1}) 3312 (ν_{N-H}, m), 3086 (ν_{O-H}, broad), 2964 (ν_{C-H}, m), 2239 ($\nu_{C\equiv N}$, w), 1674 ($\nu_{C=O}$, s), 1532, ($\nu_{C=C}$, s); HRMS (ESI): m/z calcd. for $C_{19}H_{16}FeN_2O_2$ 360.0555$[M]^+$; found $[M-CN]^+$ 360.05803; ^{1}H NMR (600 MHz, DMSO-d_6): δ (ppm) 4.27(s, 1H, H-Cp), 4.30(s, 6H, H-Cp) 4.44 (s, 1H, H-Cp), 4.48(s, 1H, H-Cp) 5.71 (d, 1H, J=8.5Hz, CNCH), 6.92 (d, 2H, J=8.7Hz, $H_{Ph2,}$), 6.99 (d, 1H, J=8.5Hz, NH), 7.81 (d, 2H, J=8.7Hz, $H_{Ph3'}$), 12.32(1H, COOH); ^{13}C NMR (150 MHz, DMSO-d_6), δ ppm: 45.1 (CH), 68.1 (C_{Cp}), 68.9 (C_{Cp}), 69.0 (C_{Cp}), 69.2 (C_{Cp}), 69.7(5C_{Cp}, overlap), 82.0 (q $C_{Cp)}$, 112.8(2$C_{2'}$), 119.47(q CN), 121.1 (q $C_{4'}$), 131.5(2$C_{3'}$), 150.1 (q $C_{1'}$), 167.8 (q C=O).

Methyl 4-((cyano(phenyl)methyl)amino)benzoate (2l)

Recrystallization from ethanol gave a yellow solid, yield: 94 % (0.31g); m.p. 128-129 ^{0}C; FT-IR (KBr, $\tilde{\upsilon}_{max}$/cm^{-1}) 3341 (ν_{N-H}, m), 2251($\nu_{C\equiv N}$, w), 1730 ($\nu_{C=O}$, s), 1120 (ν_{C-O}, s). MS (EI, 70 eV), *m/z* (%): 266$[M]^+$(100); ^{1}H NMR (400 MHz, $CDCl_3$) δ ppm: 3.88 (s, 3H, CH_3), 4.62 (d, 1H, J=7.8Hz, NH), 5.51 (d, 1H, J=7.8Hz, CH), 6.77 (d, 2H, J=8.7Hz $H_{Ph2'}$), 7.49-7.50 (m, 3H, $H_{Ph3,4}$), 7.59-7.61 (m, 2H, H_{Ph2}), 7.97(d, 2H, J=8.7Hz, $H_{Ph3'}$); ^{13}C NMR (100.5 MHz, $CDCl_3$) δ(ppm): 49.4 (OCH3), 51.8 (CNCH), 112.9(2$C_{2'}$), 117.6 (qCN), 121.4 (q $C_{4'}$), 127.2(2C_3), 129.5(2C_2), 129.8 (C_4), 131.6(2$C_{3'}$), 133.1 (q C_1), 148.4 (q$C_{1'}$), 166.9 (q C=O).

4. Crystallographic data

The details of the crystal structure determination and refinement for compound **2a** are given in Table S1.

Table S1: Crystallographic data for 2-phenothiazinyl-2-(*p*-tolylamino)acetonitrile (**2a**).

Empirical formula	$C_{22}H_{19}N_3S$
Formula weight	357.46 g/mol
Temperature	294(2) K
Wavelength	0.71073 Å
Crystal system	Orthorhombic
Space group	Pca21
a [Å]	13.948(6)
b [Å]	17.457(7)
c [Å]	7.755(3)
α [°]	90
β [°]	90
γ [°]	90
Volume	1888.3(13) $Å^3$
Z	4
Density (calculated)	1.257 g/cm^3
Absorption coefficient	0.181 mm^{-1}
F(000)	752
Crystal size	0.370 x 0.280 x 0.220 mm
Theta range for data collection	2.333 to 24.987
Index ranges	-16<=h<=16, -20<=k<=20, -9<=l<=9
Reflections collected	17118
Independent reflections	3315 [R(int) = 0.1230]
Completeness to theta =24.99°	99.8 %
Absorption correction	Semi-empirical from equivalents
Max. and min. transmission	0.961 and 0.936
Refinement method	Full-matrix least-squares on F^2
Data / restrains / parameters	3315 / 1 / 241
Goodness-of-fit on F^2 -S	1.041
Final R indices [I>2sigma(I)]	R1 = 0.0741, wR2 = 0.1451
R indices (all data)	R1 = 0.1073, wR2 = 0.1590
Largest diff. peak and hole	0.334 and -0.235 e/$Å^3$

5. Biological assay

The plate incorporation method [4] was employed for the assessment of the mutagenic and antimutagenic activity for a series of three representatives of the newly synthetized compounds (**2c, 2i, 2l**) using *S. typhimurium* TA98 and TA 100, respectively.

Bacterial strains

S. typhimurium TA98 and TA 100 bacterial strains were obtained from Food Biotechnology Laboratory, Life Sciences Institute, University of Agricultural Sciences and Veterinary Medicine Cluj-Napoca, Romania, cultured on Muller–Hinton Agar, stored at 4 °C and subcultured once a month. Sterile Erlenmeyer flasks containing 20 mL of Oxoid nutrient broth were inoculated with the tested bacteria strains and incubated at 37 °C at 150 rpm for 16 hours. The starting cell density of the bacterial cultures was $1–2\times10^9$ bacteria/mL, with absorbances at 660 nm in the range of 1.2 to 1.4.

Viability assay

The viability assays were based on a non-statistical procedure for evaluating the spontaneous mutation [5] induced by saturated solutions of the new compounds **2c, 2i**, and **2l**, respectively, in dimethyl sulfoxide (DMSO) (**2c**, 79.8 mM; **2i**, 163.6 mM; **2l**, 37.5 mM).

The positive controls were the known mutagens 2-aminoanthracene or sodium azide, NaN_3.

The negative control was DMSO.

Mutagenicity test

A mixture of 100 µL of the bacterial culture with or without 500 µL of S9 and 100 µL test compounds was poured onto the surface of minimal glucose agar plates. Viable cell colonies were scored after incubation at 37 °C for 72 h. The experiments were performed in duplicate. The mutagenic activity was assessed by the non-statistical procedure [6].

Antimutagenicity test

A mixture of 100 µL of the bacterial culture, 100 µL mutagen (2-aminoantracene, daunomycin for *S. typhimurium* TA98 or sodium azide for *S. typhimurium* TA100), with or without additional 500 µL of S9 and 100 µL test compound were poured onto minimal glucose plates and viable cells were scored after incubation at 37 °C for 72 h.

The inhibition of mutagenicity was calculated by using equation 1 [7]

$$\% \text{ Inhibition} = [1 - N_1 / N_0] \times 100, \qquad (1)$$

where

N_1 - number of revertants per plate in the presence of both, the mutagen and tested compound

N_0 - number of revertants per plate in the positive control

The antimutagenic effect was considered weak or absent (inhibition up to 25%), moderate (25–40% inhibition) or strong (inhibition higher than 40%) [8]

References

1. Gal, E.; Găină, L.; Cristea, C.; Munteanu, V.; Silaghi-Dumitrescu, L. *J. Electroanal. Chem*. **2016**, *770*, 14-22. doi: 10.1016/j.jelechem.2016.03.019

2. Sheldrick, G. M.; SHELX-97, Universität Göttingen, Germany, **1997**.

3. DIAMOND – Visual Crystal Structure Information System, CRYSTAL IMPACT: Bonn, Germany, **2001**.

4. Maron, D.M.; Ames, B.N. *Mutat. Res.* **1983**, *113* (3/4), 173–215. doi: 10.1016/0165-1161(83)90010-9

5. Mortelmans, K.; Zeiger, E. *Mutat. Res*. **2000**, *455* (1/2), 29–60. doi: 10.1016/S0027-5107(00)00064-6

6. Zeiger, E.; Anderson, B.; Haworth, S.; Lawlor, T.; Mortelmans, K. *Environ. Mol. Mutagen*. **1992**, *19* (Suppl. 21), 1–141. doi: 10.1002/em.2850190603

7. Ong, T.; Whong, W. Z.; Stewart, J.; Brockman H. E. *Mutat. Res*. **1986**, *173*, 111-115. doi: 10.1016/0165-7992(86)90086-2

8. Evandri, M. G.; Battinelli, L.; Daniele, C.; Mastrangelo, S.; Bolle, P.; Mazzanti, G. *Food Chem. Toxicol*. *2005*, *43* (9), 1381–1387. doi: 10.1016/j.fct.2005.03.013

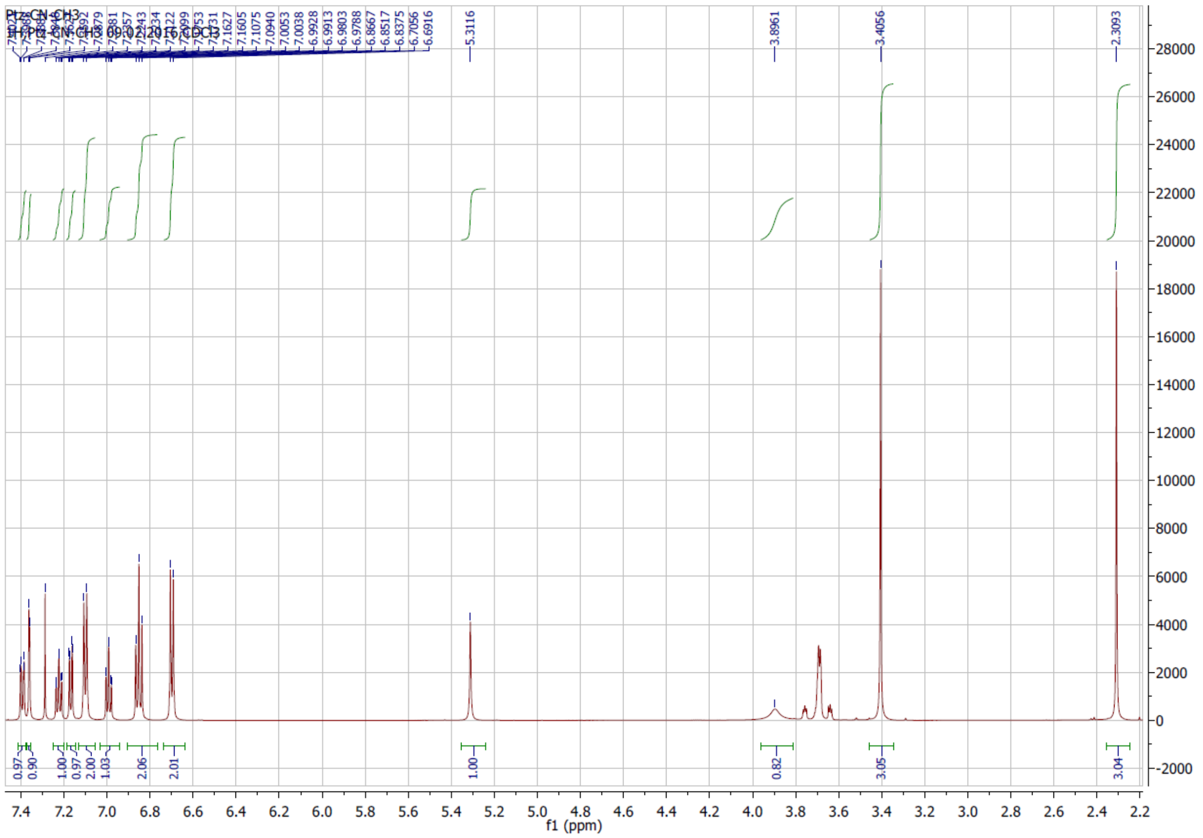

Figure S1. ^{1}H NMR spectrum (600 MHz) for compound **2a** in $CDCl_3$.

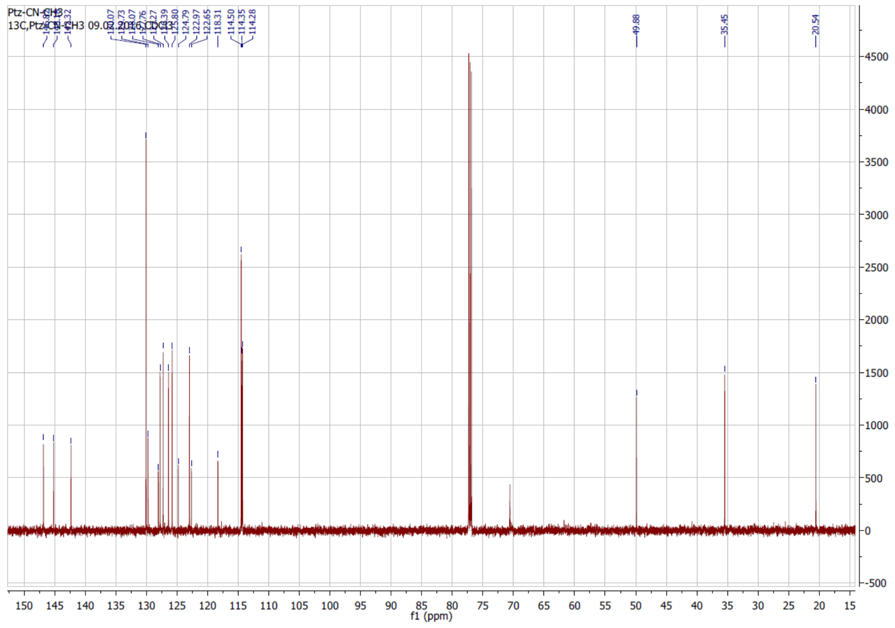

Figure S2.^{13}C NMR spectrum (150 MHz) for compound **2a** in $CDCl_3$.

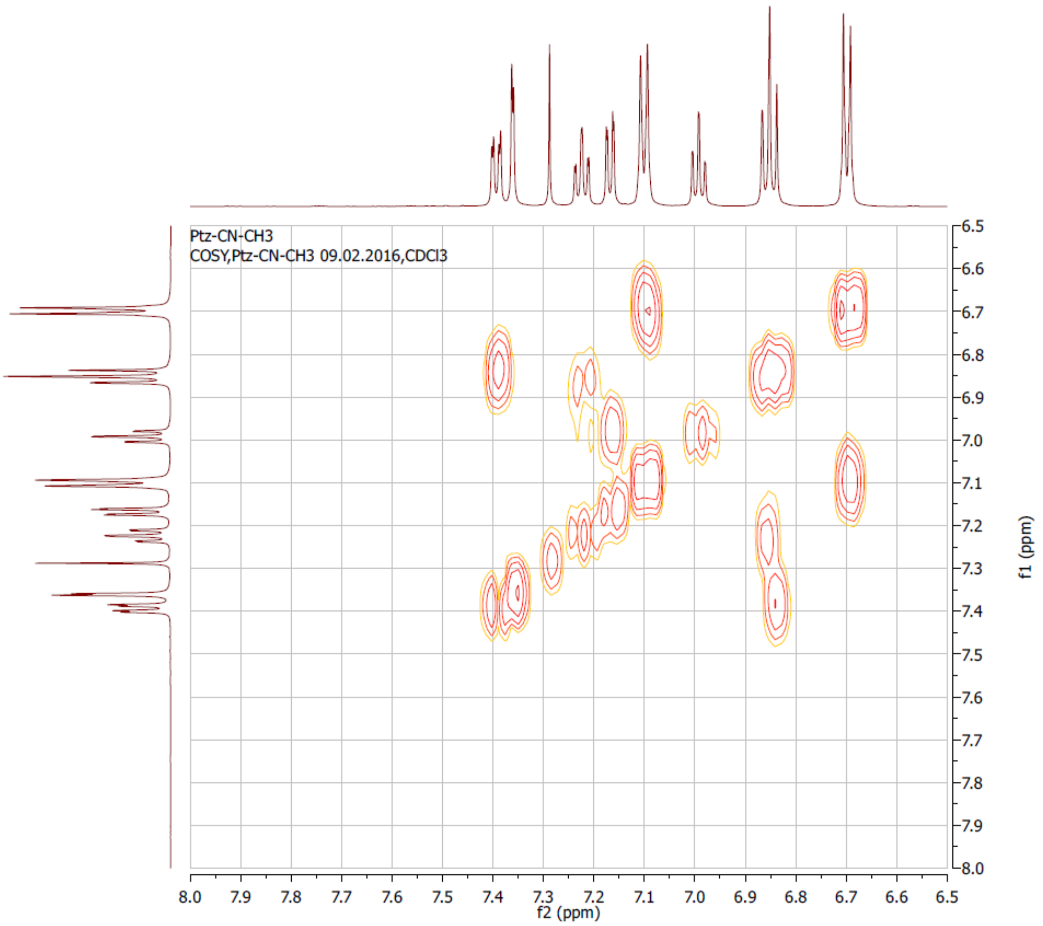

Figure S3. 2D-NMR $^{1}H/^{1}H$ COSY spectrum for compound **2a** in $CDCl_3$.

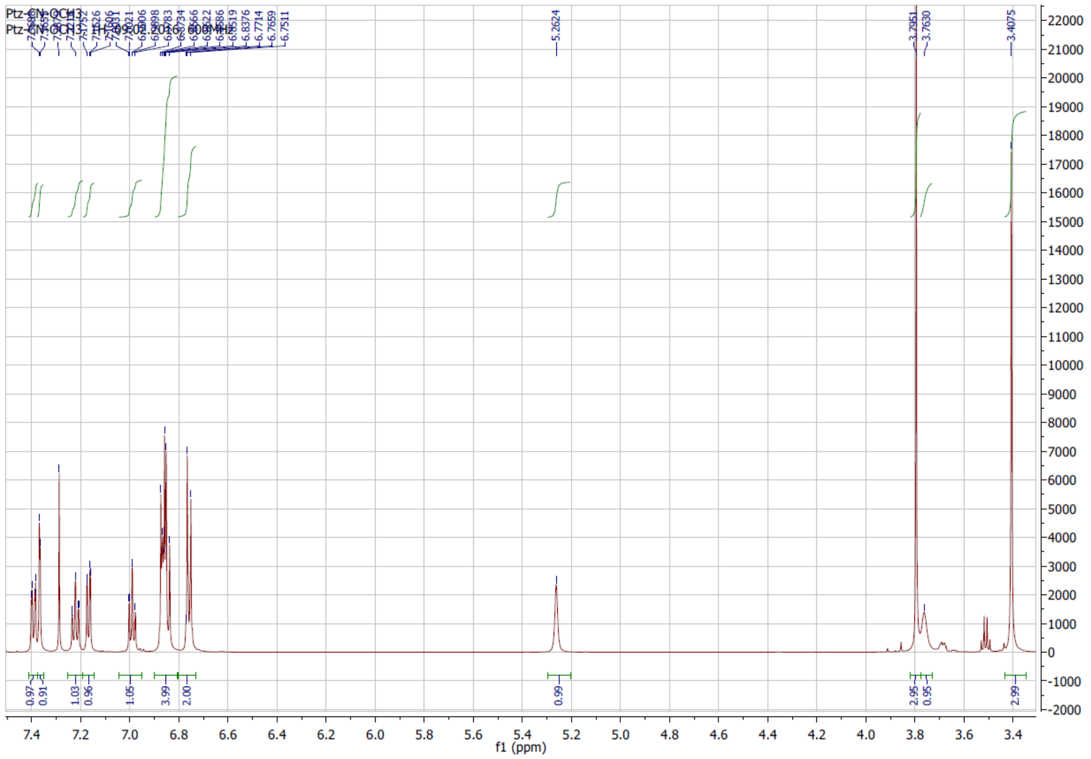

Figure S4. ^{1}H NMR spectrum (600 MHz) for compound **2b** in $CDCl_3$.

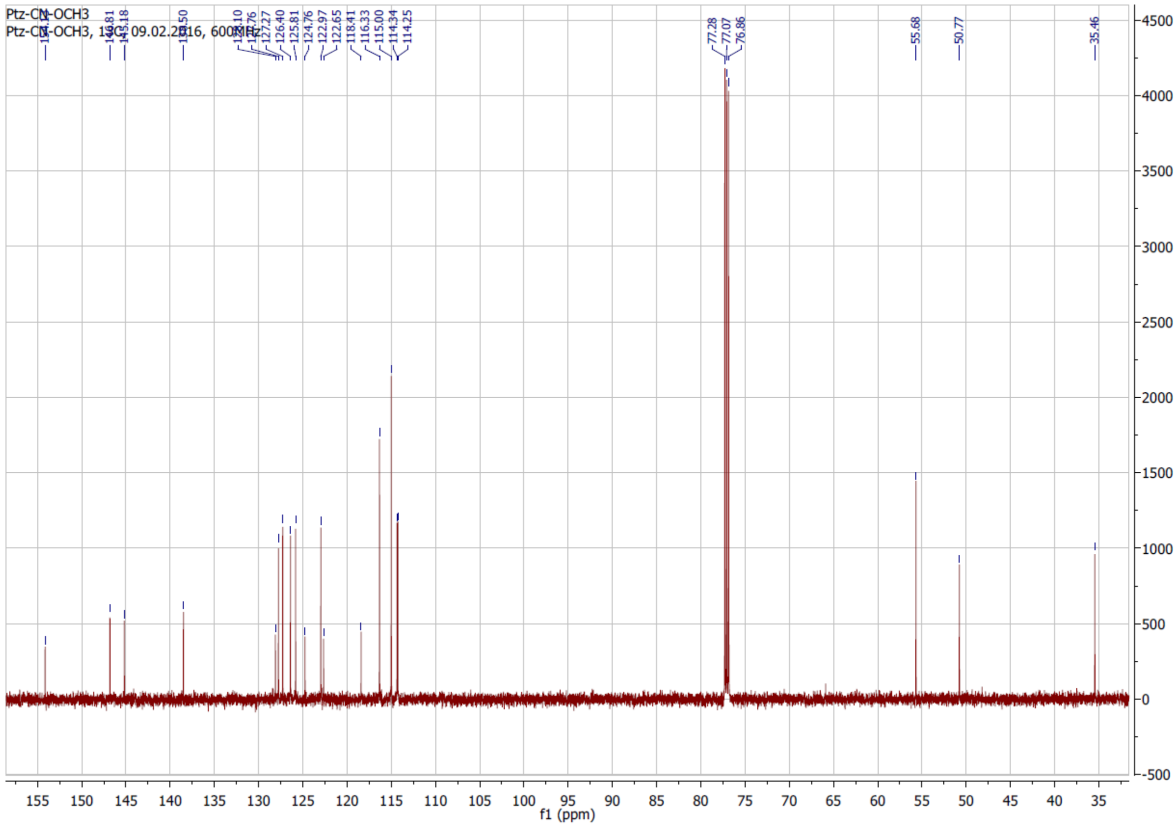

Figure S5.^{13}C NMR spectrum (150 MHz) for compound **2b** in $CDCl_3$.

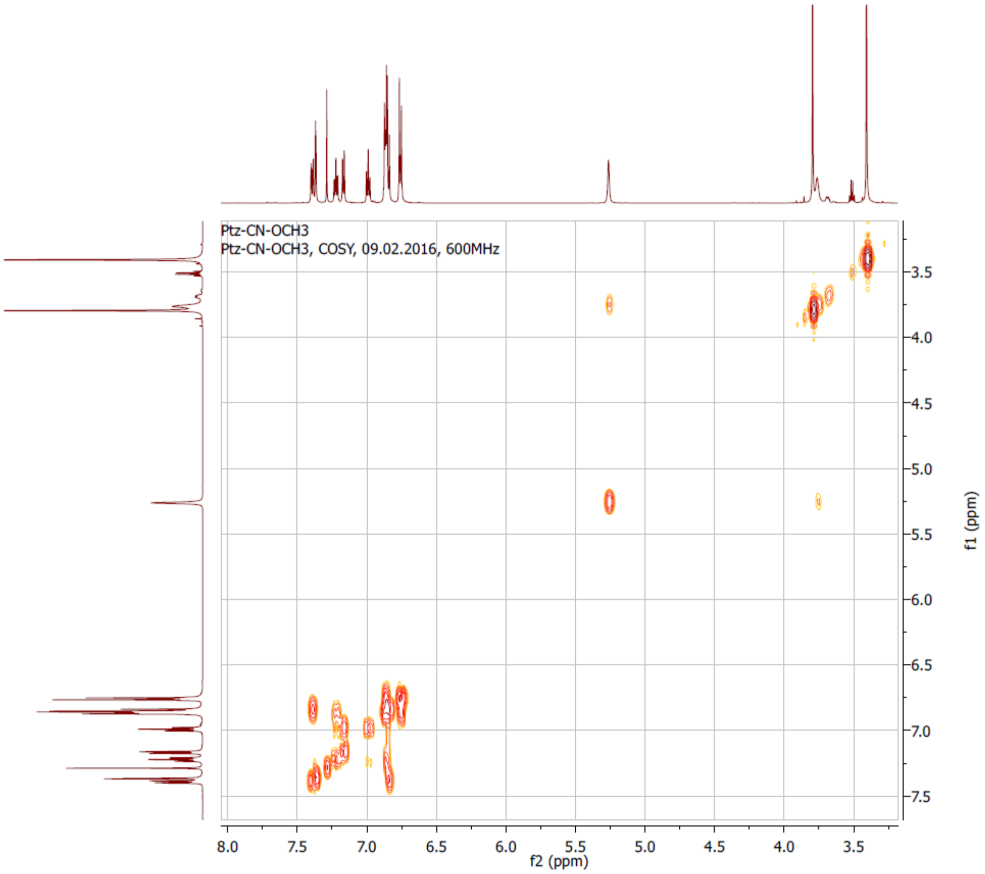

Figure S6. 2D-NMR $^{1}H/^{1}H$ COSY spectrum for compound **2b** in $CDCl_3$.

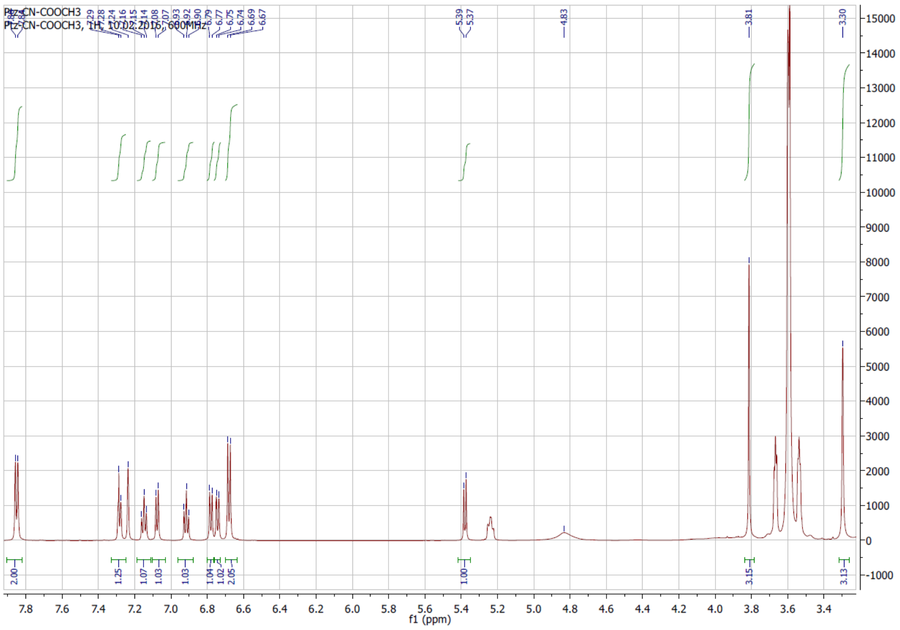

Figure S7. ^{1}H NMR spectrum (600 MHz) for compound **2c** in $CDCl_3$.

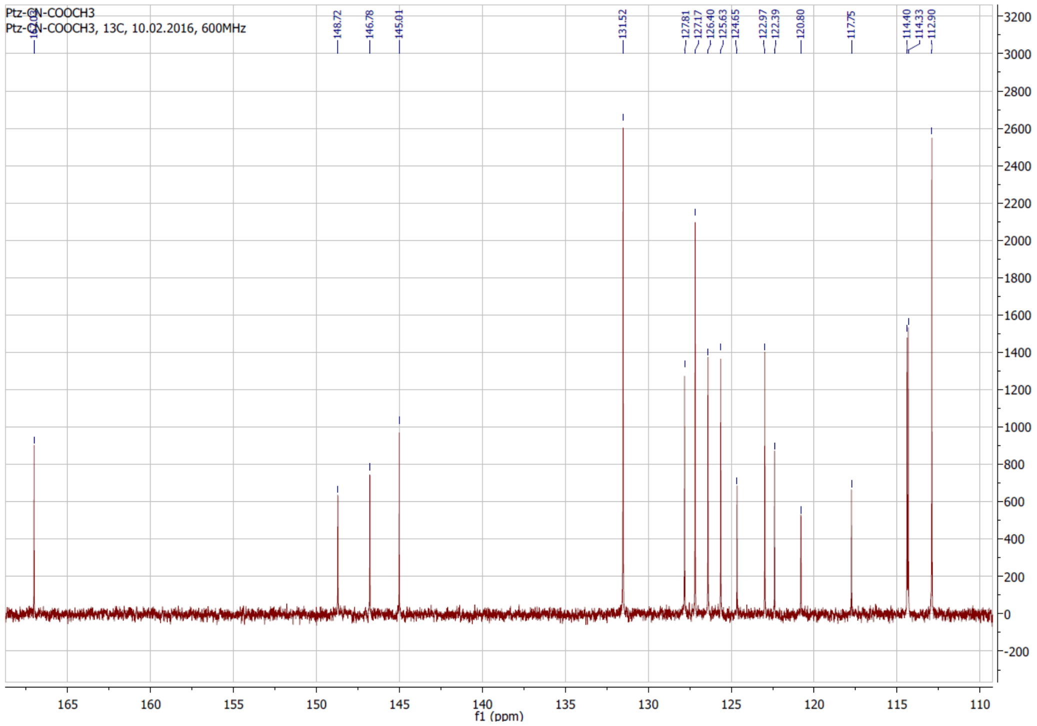

Figure S8.^{13}C NMR spectrum (150 MHz) for compound **2c** in $CDCl_3$.

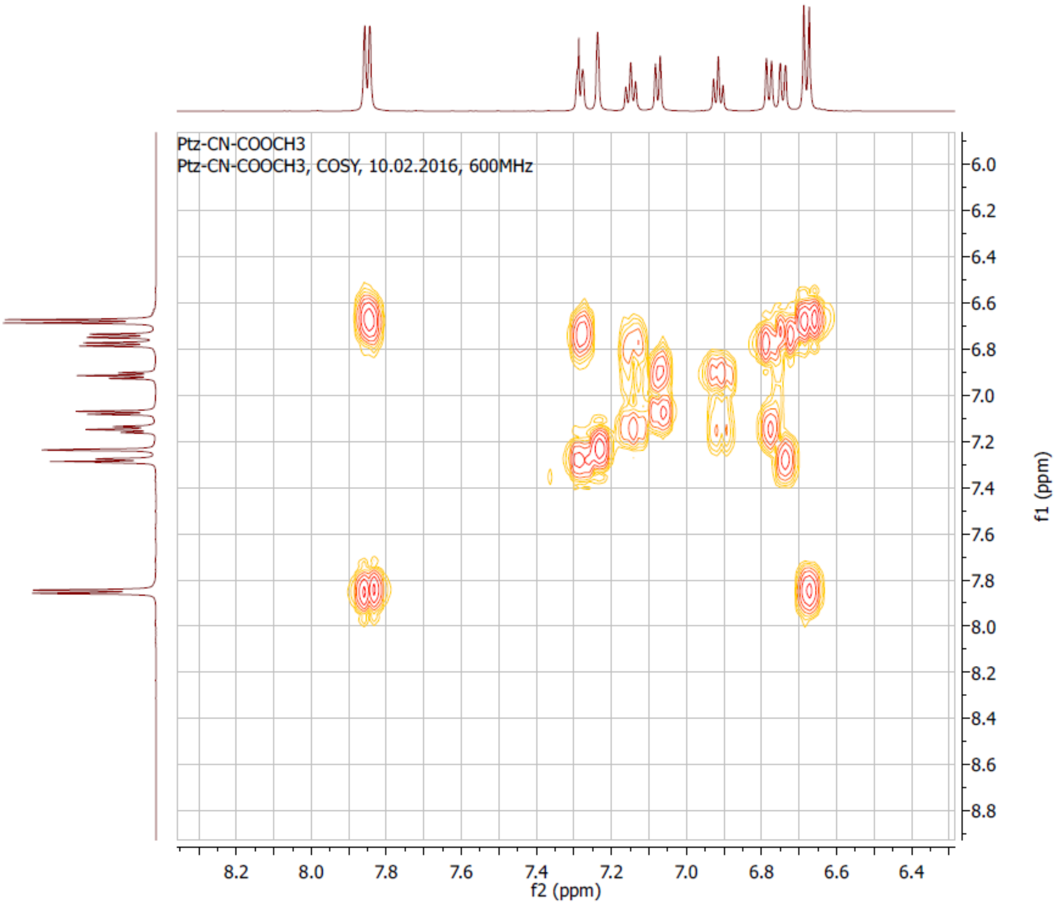

Figure S9. 2D-NMR $^1H/^1H$ COSY spectrum for compound **2c** in $CDCl_3$.

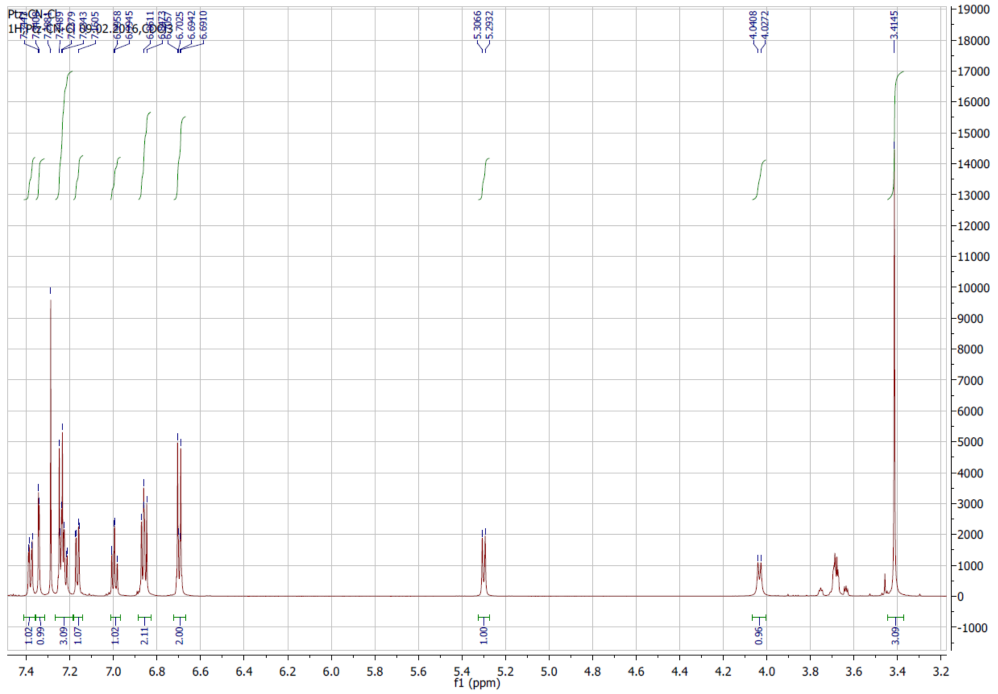

Figure S10. ^{1}H NMR spectrum (600 MHz) for compound **2e** in $CDCl_3$.

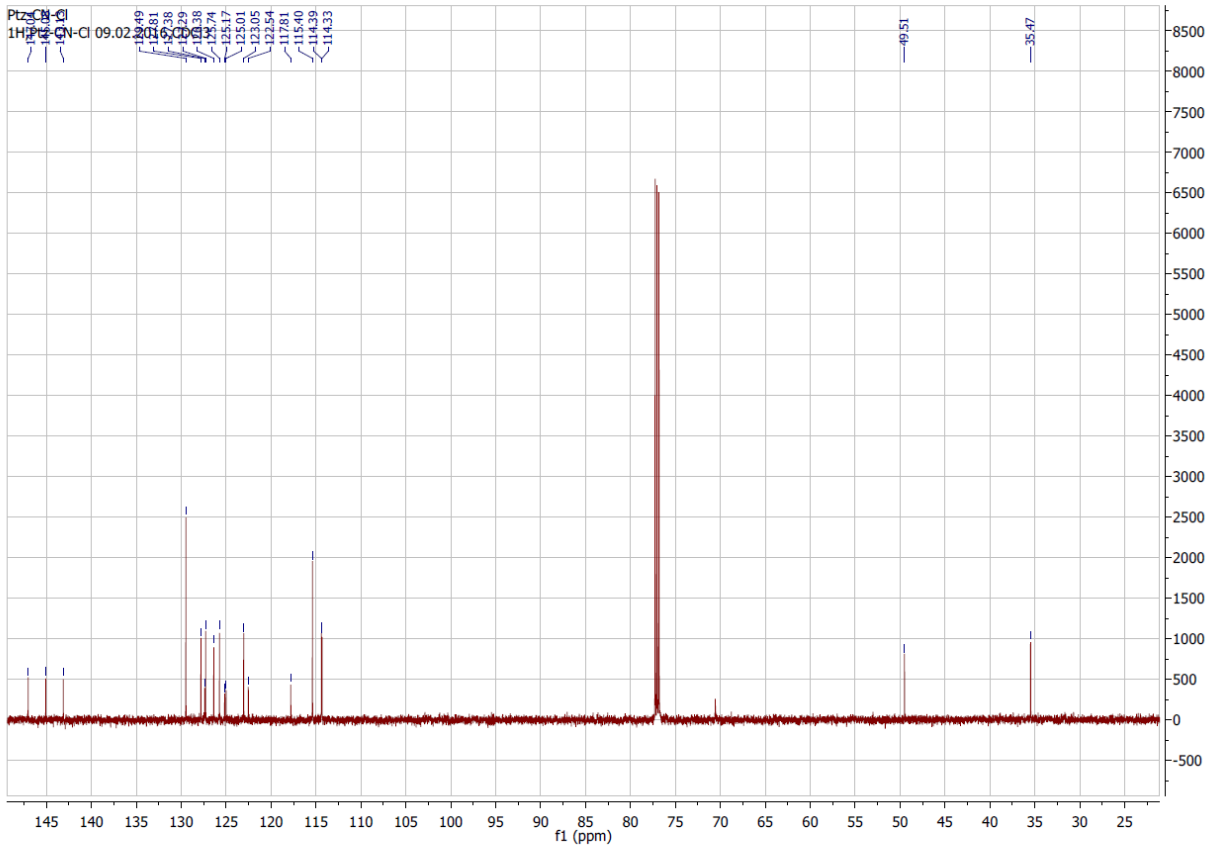

Figure S11.^{13}C NMR spectrum (150 MHz) for compound **2e** in $CDCl_3$.

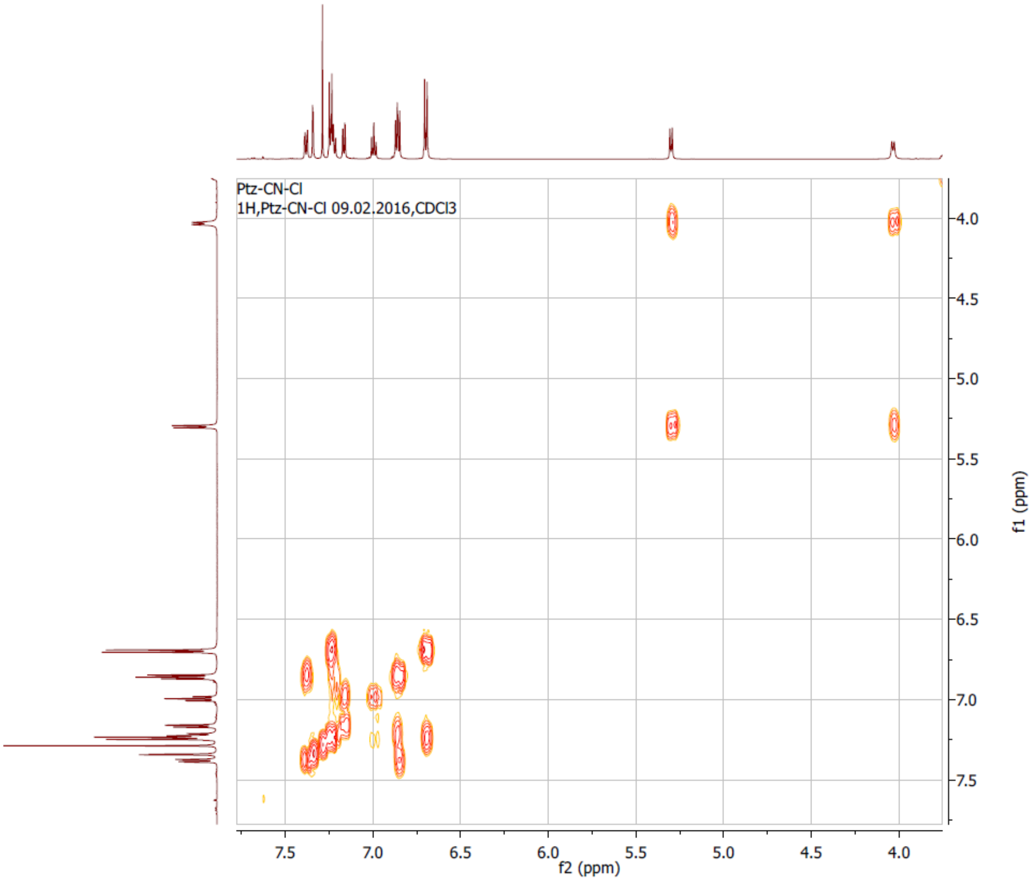

Figure S12. 2D-NMR $^{1}H/^{1}H$ COSY spectrum for compound **2e** in $CDCl_3$.

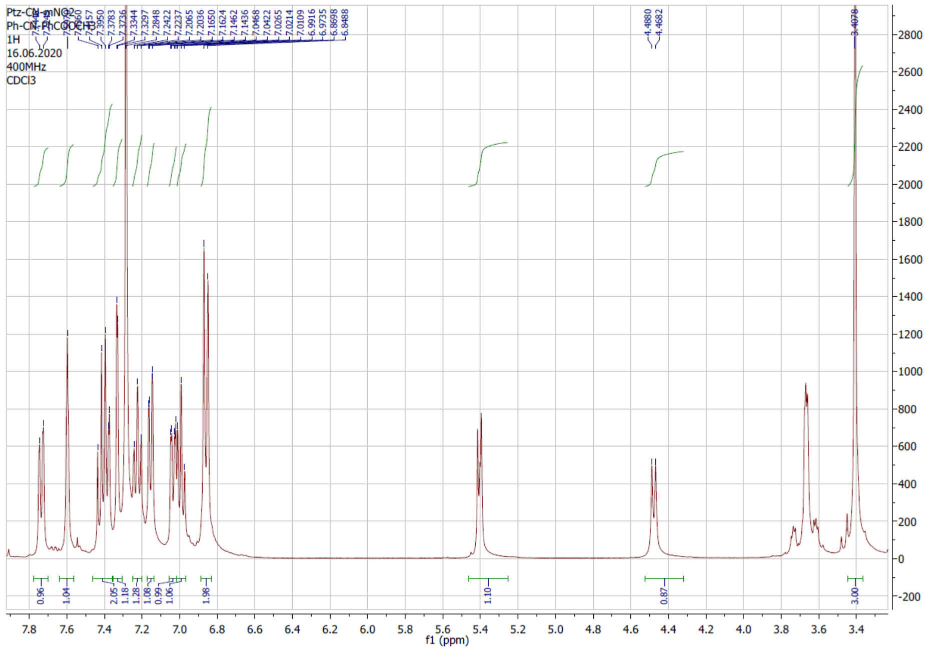

Figure S13. ^{1}H NMR spectrum (400 MHz) for compound **2g** in $CDCl_3$.

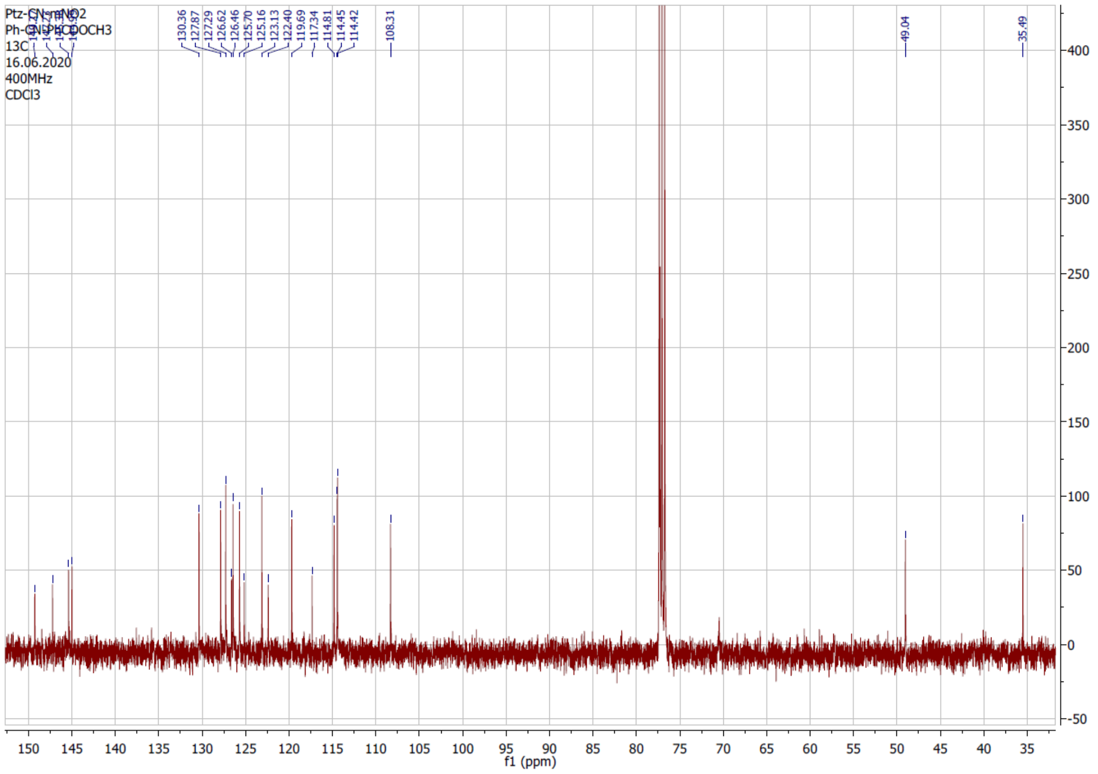

Figure S14. ^{13}C NMR spectrum (125 MHz) for compound **2g** in $CDCl_3$.

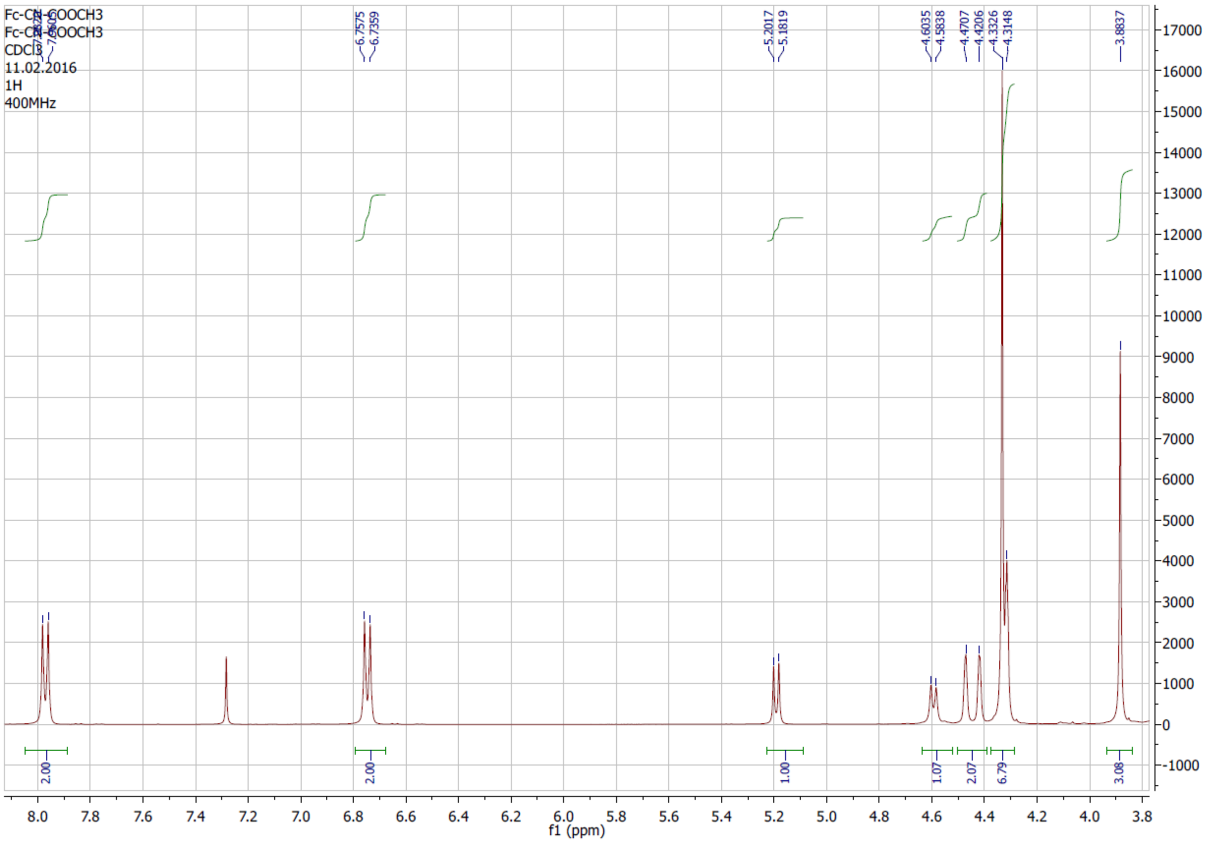

Figure S15. ^{1}H NMR spectrum (400 MHz) for compound **2i** in $CDCl_3$.

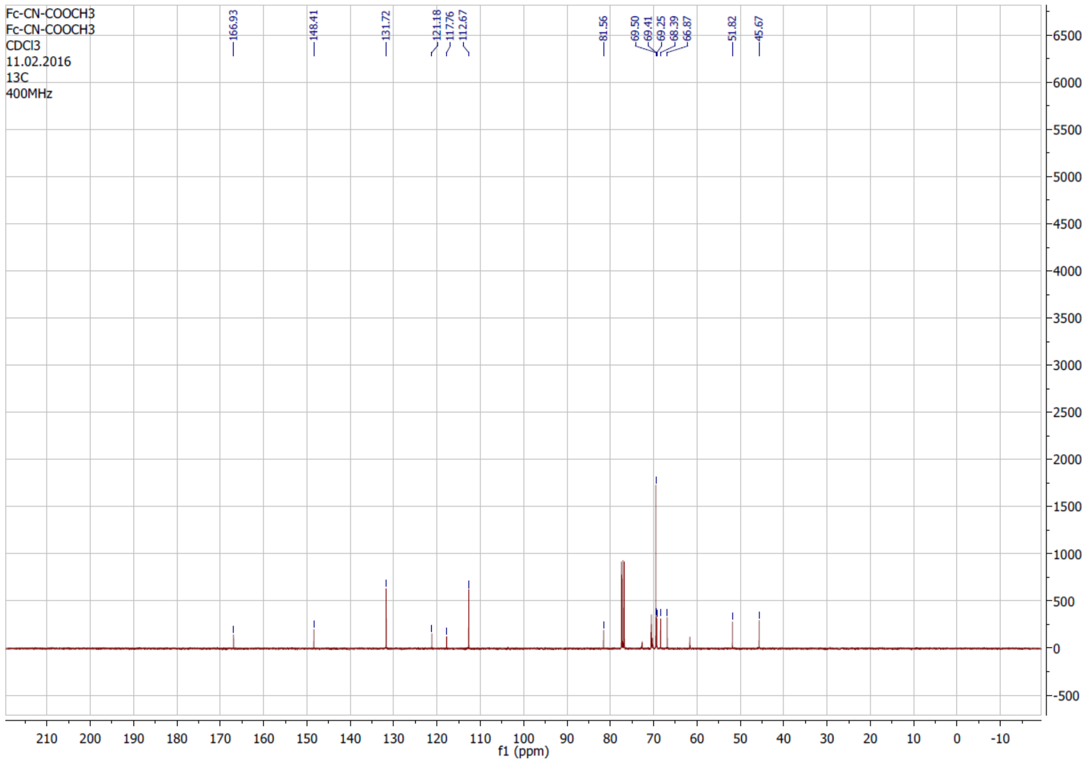

Figure S16. ${}^{13}C$ NMR spectrum (125 MHz) for compound **2i** in $CDCl_3$.

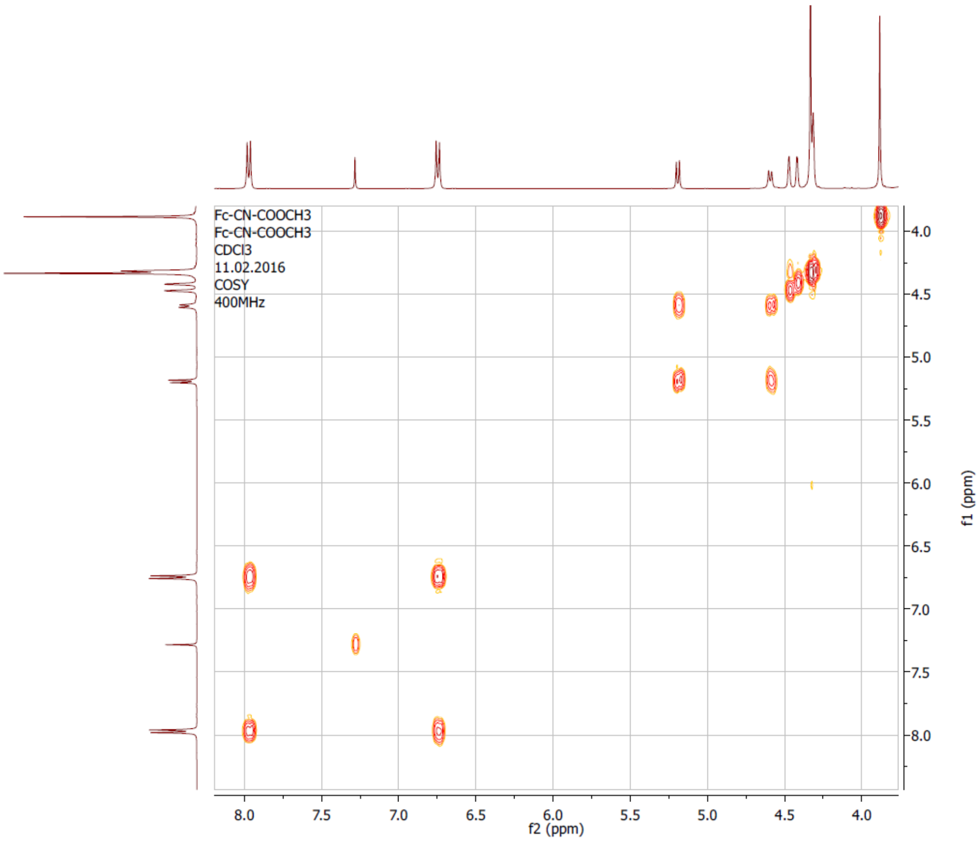

Figure S17. 2D-NMR ${}^{1}H/{}^{1}H$ COSY spectrum for compound **2i** in $CDCl_3$.

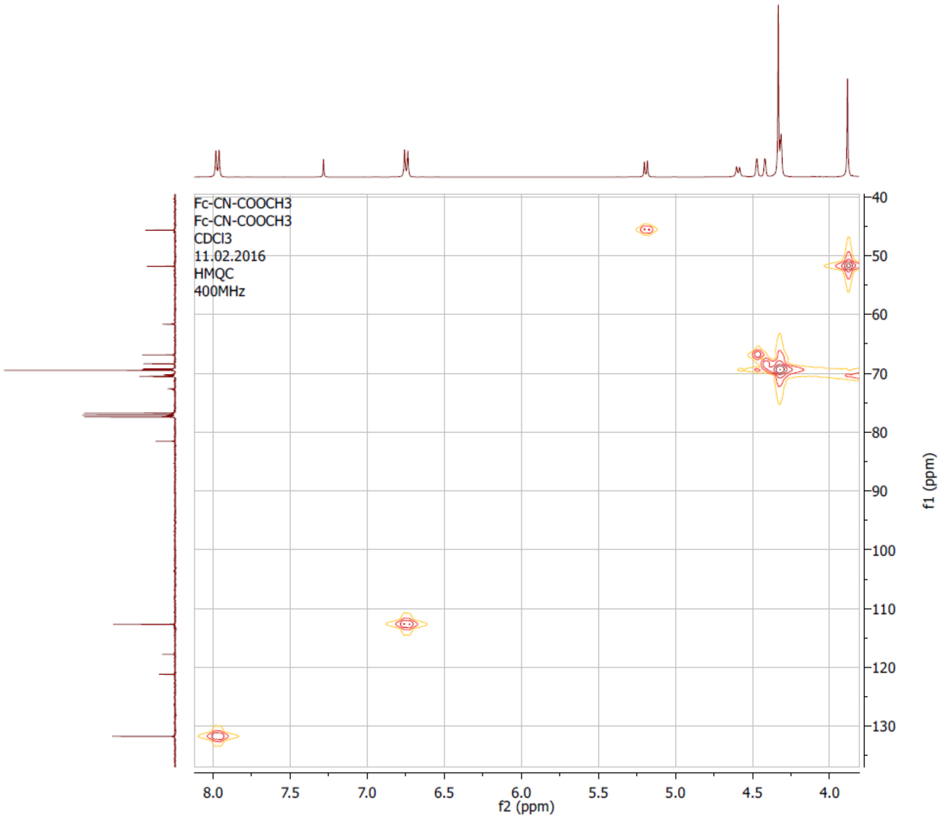

Figure S18. 2D-NMR $^{1}H/^{13}C$ HMQC spectrum for compound **2i** in $CDCl_3$.

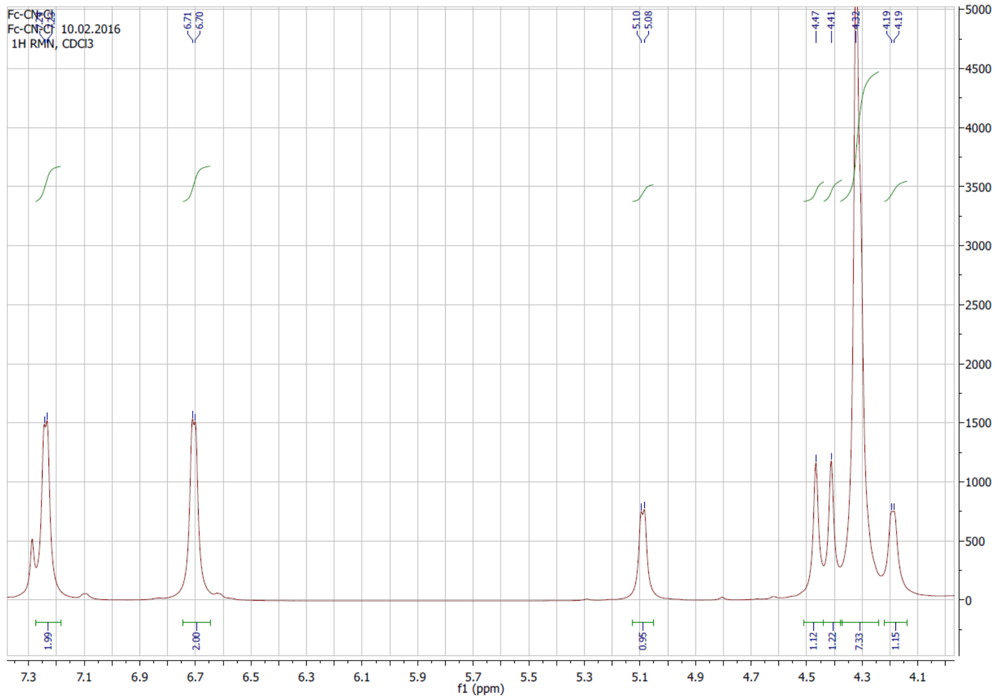

Figure S19. ^{1}H NMR spectrum (600 MHz) for compound **2j** in $CDCl_3$.

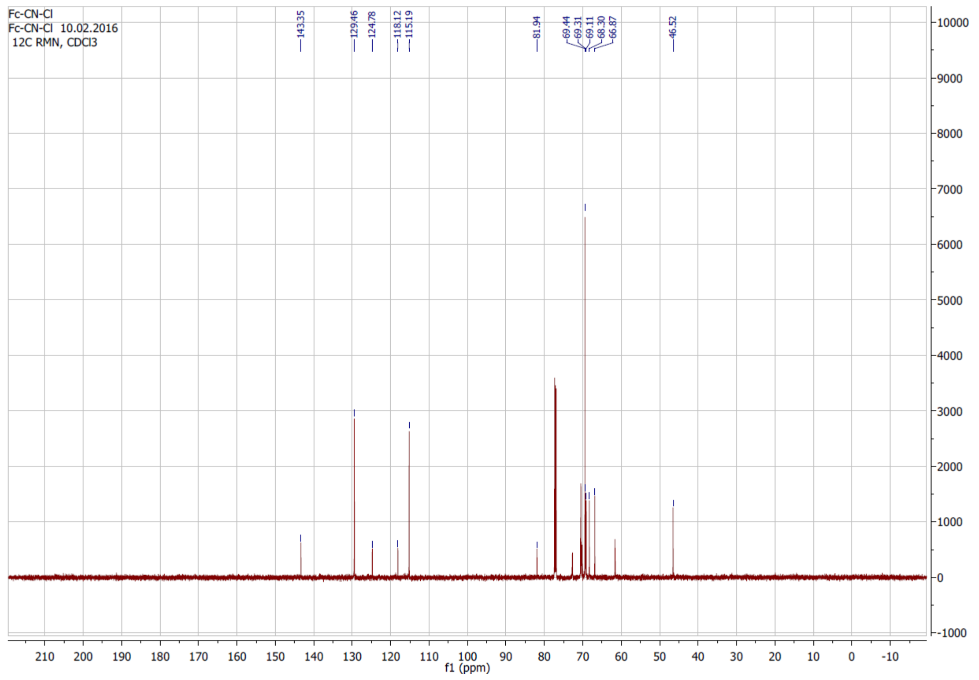

Figure S20. ^{13}C NMR spectrum (150 MHz) for compound **2j** in $CDCl_3$.

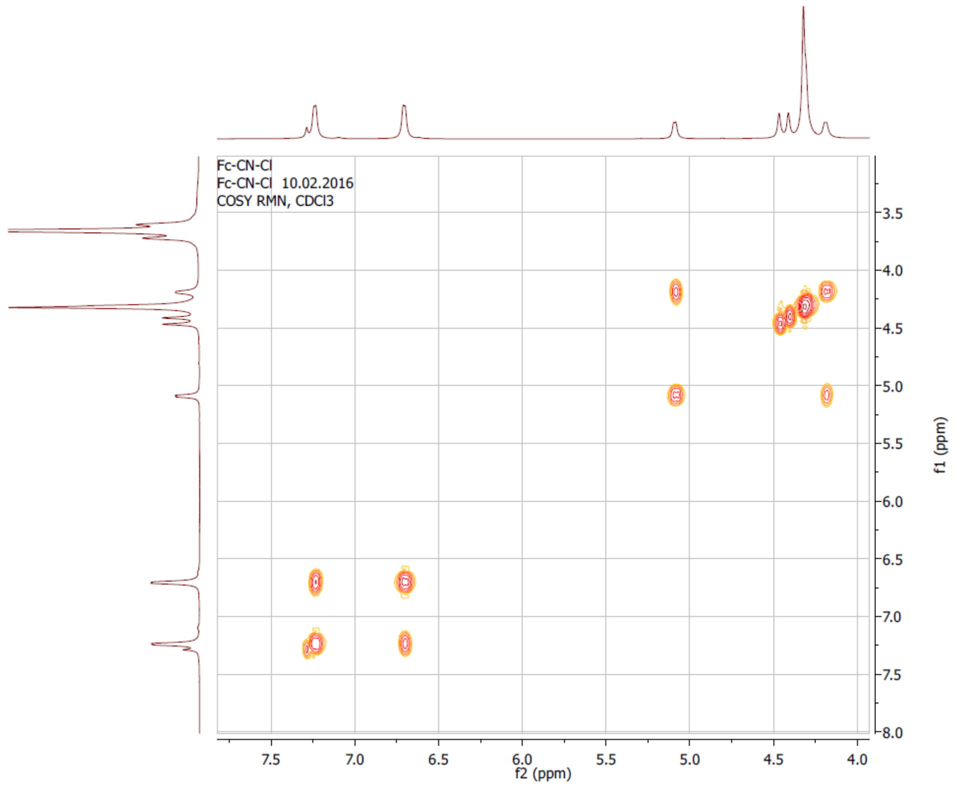

Figure S21. 2D-NMR ^{1}H/^{1}H COSY spectrum for compound **2j** in $CDCl_3$.

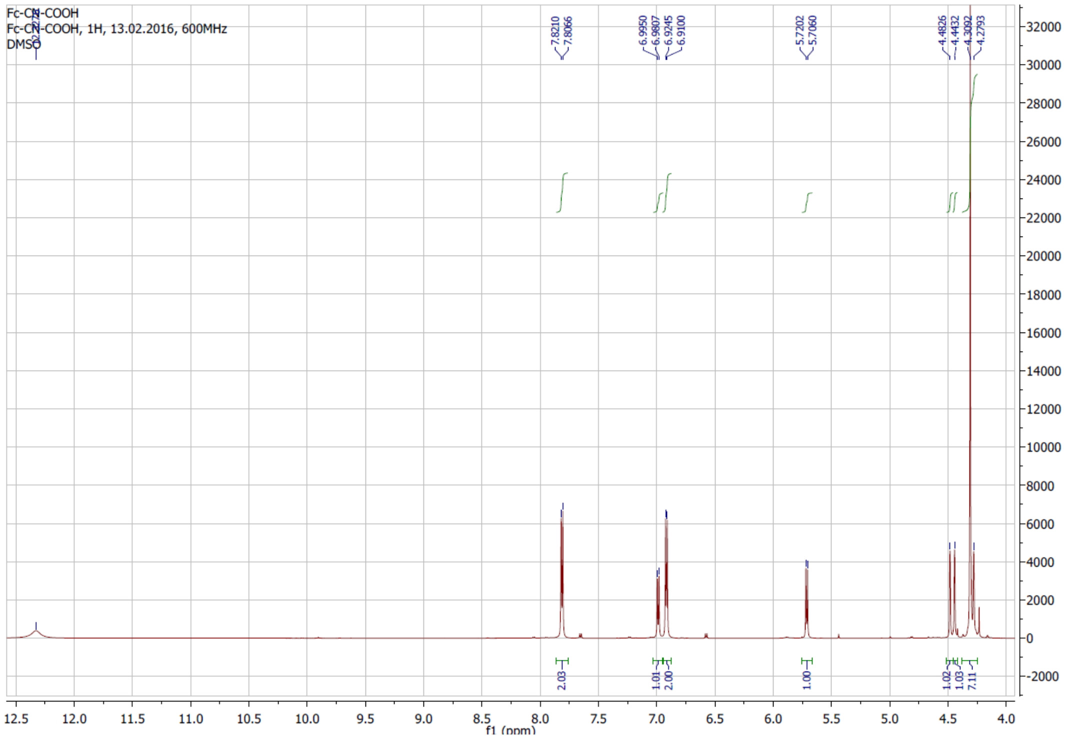

Figure S22. ^{1}H NMR spectrum (600 MHz) for compound **2k** in DMSO-d_6.

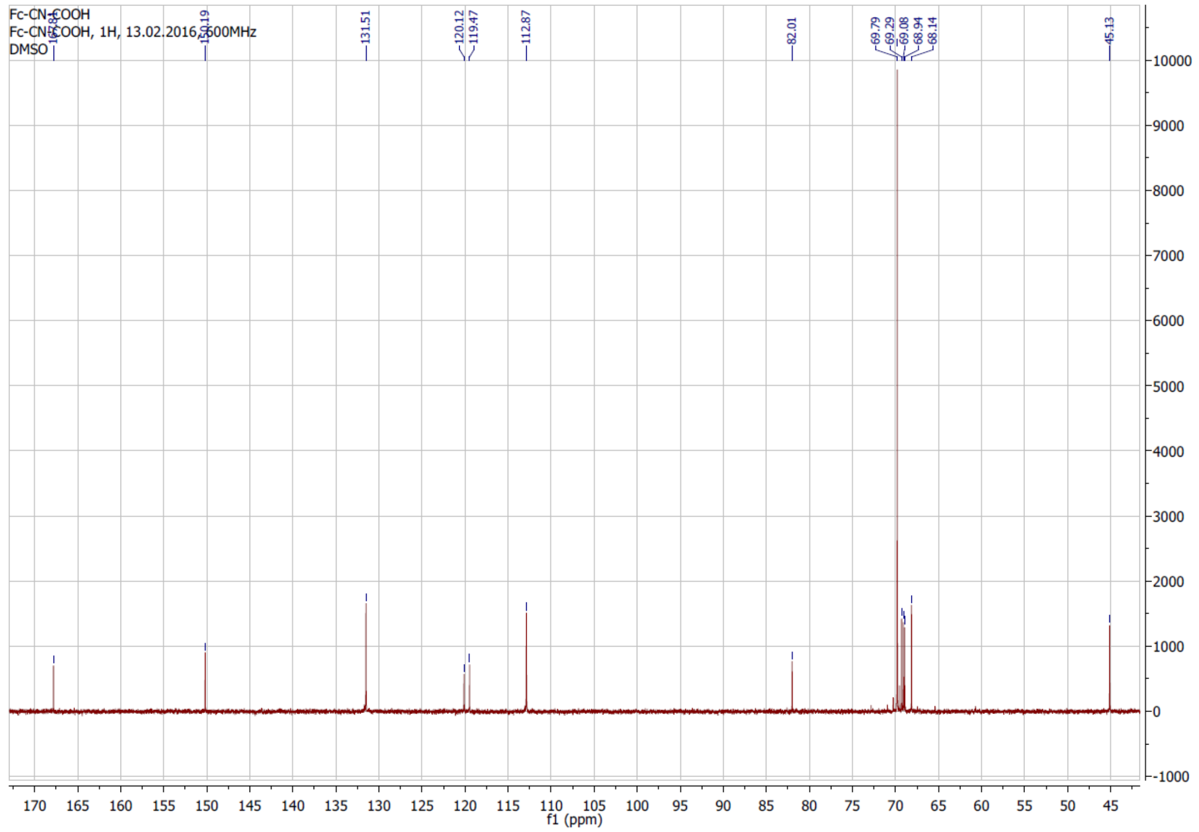

Figure S23. ^{13}C NMR spectrum (150 MHz) for compound **2k** in DMSO-d_6.

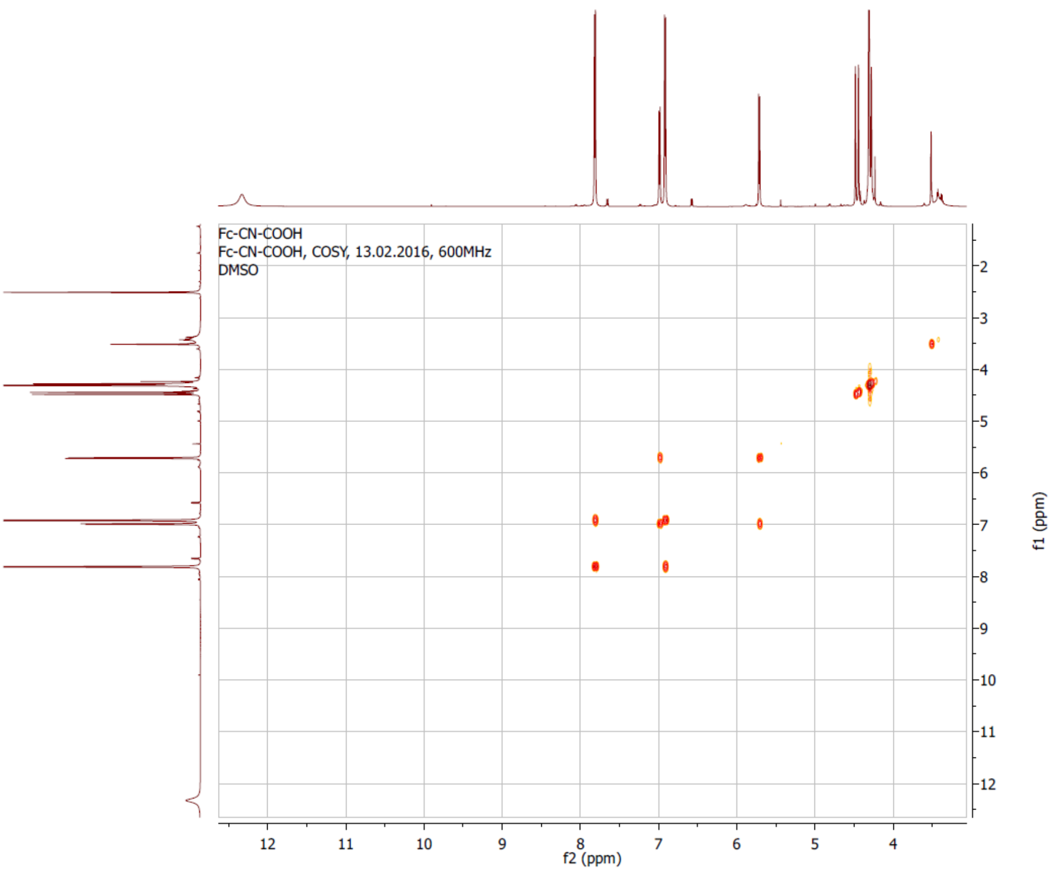

Figure S24. 2D-NMR $^{1}H/^{1}H$ COSY spectrum for compound **2k** in DMSO-d_6.

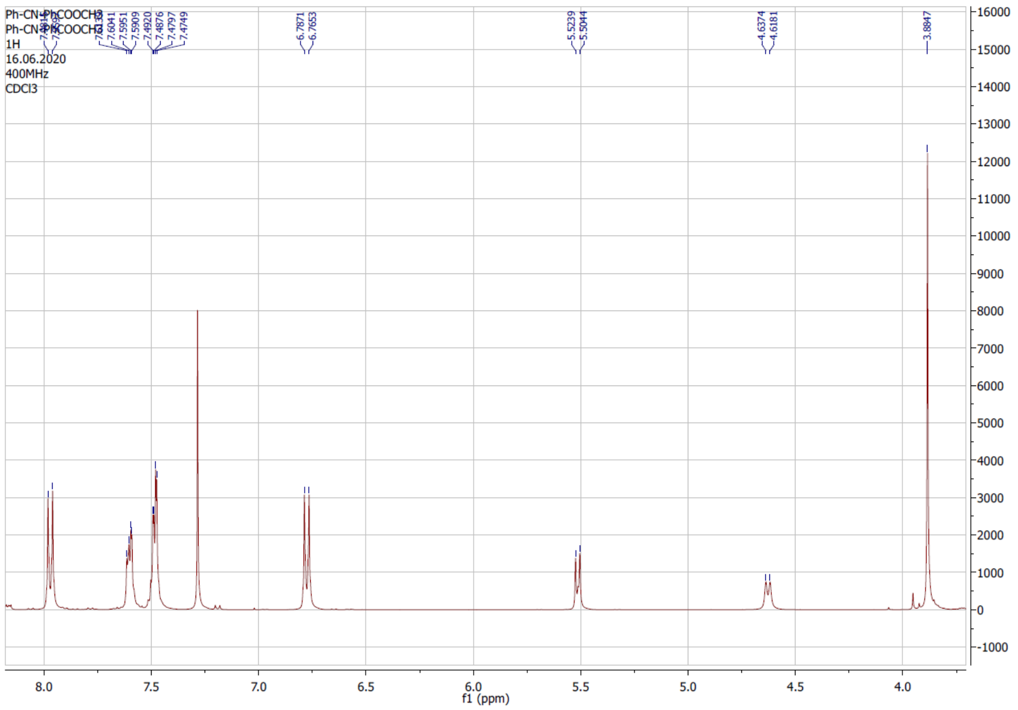

Figure S25. ^{1}H NMR spectrum (400 MHz) for compound **2l** in $CDCl_3$.

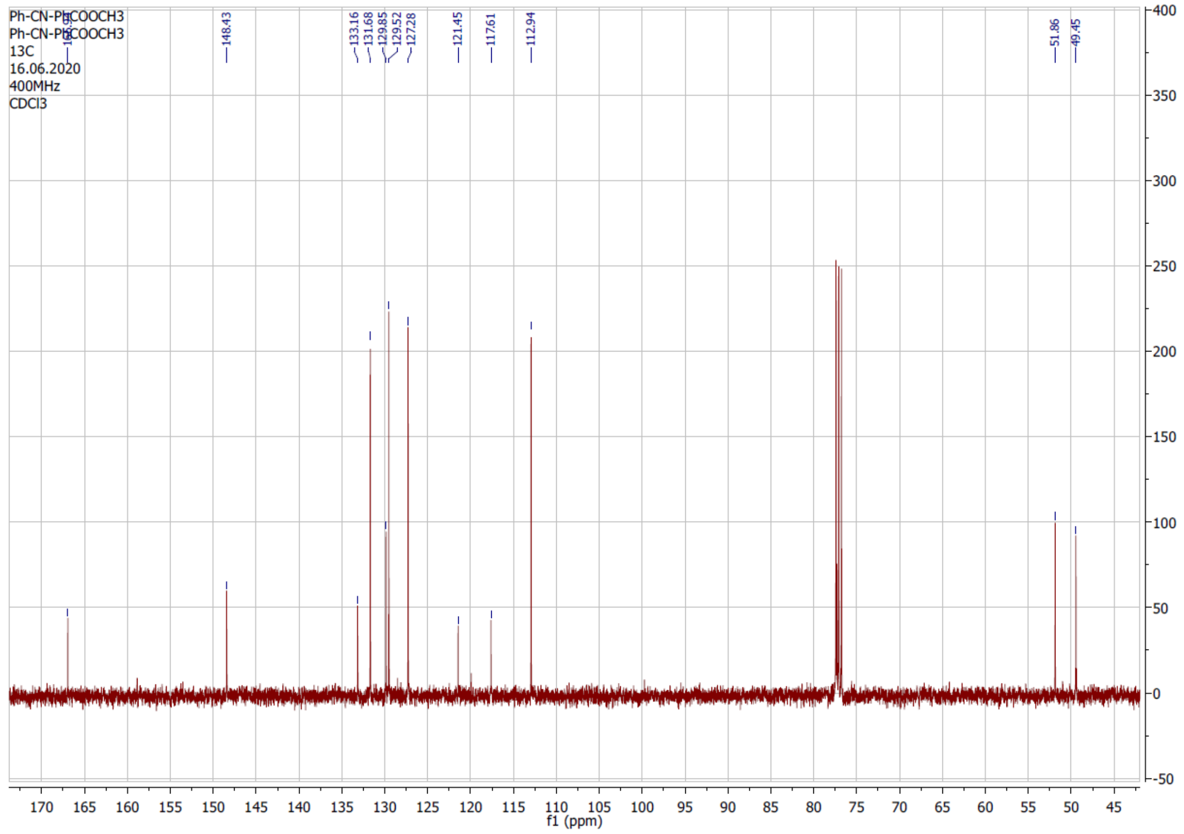

Figure S26. ^{13}C NMR spectrum (125 MHz) for compound **2l** in $CDCl_3$.
